# Intestinal-derived FGF15 protects against deleterious effects of vertical sleeve gastrectomy in mice

**DOI:** 10.1038/s41467-021-24914-y

**Published:** 2021-08-06

**Authors:** Nadejda Bozadjieva-Kramer, Jae Hoon Shin, Yikai Shao, Ruth Gutierrez-Aguilar, Ziru Li, Kristy M. Heppner, Samuel Chiang, Sara G. Vargo, Katrina Granger, Darleen A. Sandoval, Ormond A. MacDougald, Randy J. Seeley

**Affiliations:** 1 Department of Surgery, University of Michigan, Ann Arbor, MI USA; 2 Center for Obesity and Metabolic Surgery, Huashan Hospital of Fudan University, Shanghai, China; 3 Divsión de Investigación, Facultad de Medicina, Universidad Nacional Autónoma de México and Laboratorio de Enfermedades Metabólicas: Obesidad y Diabetes, Hospital Infantil de México “Federico Gómez”, Mexico, Mexico; 4 Department of Molecular & Integrative Physiology, University of Michigan, Ann Arbor, MI USA; 5 Novo Nordisk Research Center Seattle, Inc, Seattle, WA USA; 6 Department of Pediatrics, Section of Nutrition, University of Colorado Anschutz Medical Campus, Aurora, CO USA

**Keywords:** Bone quality and biomechanics, Metabolic diseases, Obesity, Gastrointestinal hormones

## Abstract

Bariatric surgeries such as the Vertical Sleeve Gastrectomy (VSG) are invasive but provide the most effective improvements in obesity and Type 2 diabetes. We hypothesized a potential role for the gut hormone Fibroblast-Growth Factor 15/19 which is increased after VSG and pharmacologically can improve energy homeostasis and glucose handling. We generated intestinal-specific FGF15 knockout (FGF15^INT-KO^) mice which were maintained on high-fat diet. FGF15^INT-KO^ mice lost more weight after VSG as a result of increased lean tissue loss. FGF15^INT-KO^ mice also lost more bone density and bone marrow adipose tissue after VSG. The effect of VSG to improve glucose tolerance was also absent in FGF15^INT-KO^. VSG resulted in increased plasma bile acid levels but were considerably higher in VSG-FGF15^INT-KO^ mice. These data point to an important role after VSG for intestinal FGF15 to protect the organism from deleterious effects of VSG potentially by limiting the increase in circulating bile acids.

## Abstract (web summary)

The mechanisms that mediate the effects of weight loss surgeries such as vertical sleeve gastrectomy (VSG) are incompletely understood. Here the authors show that intestinal FGF15 is necessary to improve glucose tolerance and to prevent the loss of muscle and bone mass after VSG, potentially via protection against bile acid toxicity.

## Introduction

Obesity has become a growing epidemic, where associated complications such as cardiovascular morbidity, Type 2 diabetes, and insulin resistance pose major health care challenges^1^. Current pharmacological treatments for obesity include <10 FDA-approved drugs, all of which demonstrate modest effect sizes^2^. Although invasive, bariatric surgery is the most effective treatment for sustained weight loss and also improves glycemic control better than conventional weight-loss therapies^3–5^.

The gastrointestinal tract plays an important role in regulating many of the potent metabolic effects of bariatric surgery leading to reduced body weight and improved glucose metabolism. An important weight-independent effect of bariatric surgery is the alteration of enterohepatic bile acid circulation resulting in increased plasma bile levels as well as altered bile acid composition in rodents^6,7^ and humans^8,9^. We and others have hypothesized that changes in the enterohepatic metabolism play important role in the potent metabolic effects of vertical sleeve gastrectomy (VSG) and Roux-Y Gastric Bypass (RYGB)^10,11^. We identified bile acid signaling through the nuclear ligand-activated farnesoid X receptor (FXR) as a potential link for mediating the beneficial effects of elevated bile acids following bariatric surgery. We also reported that bile acids are increased after VSG and that FXR is essential for the positive effects of bariatric surgery on weight loss and glycemic control^6,12^. Unlike wild-type mice, *FXR−/−* mice do not maintain body weight loss and do not have improved glucose tolerance after VSG or after bile diversion to the ileum^12,13^. These studies highlighted the importance of enterohepatic circulation in the metabolic effects following bariatric surgery.

Absorbed bile acids activate intestinal FXR and its downstream target FGF15/19 (mouse/human ortholog, respectively). FGF15/19 is a gut hormone expressed in ileal enterocytes of the small intestine and is released postprandially in response to bile acid absorption among other stimuli^14–17^. Once released from the ileum, FGF15/19 enters the portal venous circulation and travels to the liver where FGF15/19 binds to its receptor FGFR4 and represses de novo bile acid synthesis and gallbladder filling^14^. Pharmacological administration of FGF15/19 has potent effects to reduce bile acid levels and secretion at the level of both the liver and the gallbladder^18,19^. Importantly, pharmacological administration of FGF19 reduces body weight and improves glucose regulation in rodents^20^. FGF15/19 stimulates protein and glycogen synthesis, while reducing gluconeogenesis, hepatic triglycerides, and cholesterol^18,19^. FGF19 levels also rise 2–4 h postprandially, which is considerably later than insulin and GLP-1^17^. These notable metabolic actions and protein engineering has made FGF19 analogues attractive candidates for the treatment of type 2 diabetes and hepatic lipid disorders with a number of clinical trials under way^10,21–23^.

Pharmacologically elevating FGF15/19 levels in rodent models of metabolic disease results in multiple metabolic benefits including increased energy expenditure, reduced adiposity, and improved lipid and glucose homeostasis^24–28^. FGF19 levels are lower in patients with obesity, without a strong association to glucose metabolism nor insulin sensitivity^29–33^. However, other studies report that basal FGF19 levels are inversely correlated to glucose metabolism or insulin sensitivity^34,35^ and nonalcoholic fatty liver disease (NAFLD)^36,37^. Most importantly, fasting and postprandial plasma FGF19 levels in humans^22,32,33,38–40^ and ileal FGF15 expression in mice (as shown in the present studies) increase after VSG. These data point to FGF15/19 as a potential target to mediate VSG’s effects to produce sustained weight loss and improved glucose tolerance. To test the hypothesis of whether FGF15 plays a role in the metabolic improvements after bariatric surgery, we generated a novel mouse model of intestinal-specific FGF15 knock out (VilCreERT2; Fgf15^f/f^) and controls, which were maintained on a 60% high-fat diet (HFD) before and after undergoing Sham or VSG surgery. Here we report that intestinal-derived FGF15 is necessary to prevent excessive bile acid increases and prevent the consequent loss in muscle and bone mass, the improvement in peripheral blood glucose regulation, and the decrease in hepatic cholesterol after VSG. These findings point to an important role for FGF15 in the regulation of multiple metabolic parameters following VSG.

## Results

### Intestinal FGF15 expression increases and prevents muscle mass loss after VSG in mice

**Fig. 1:**
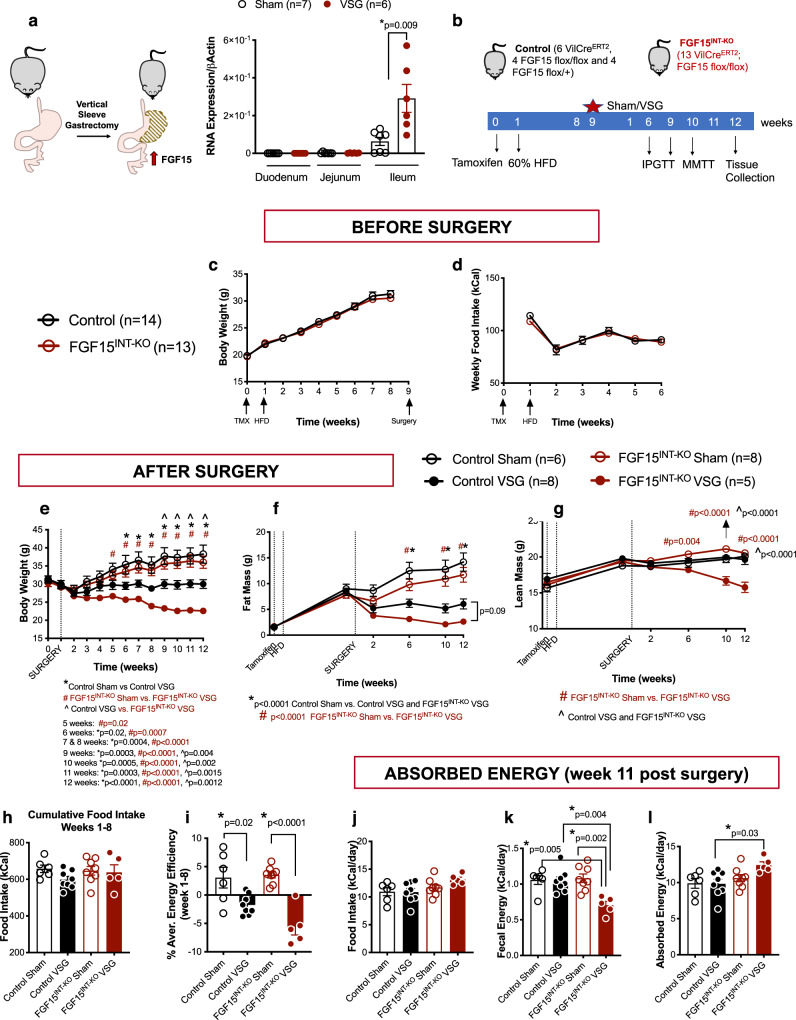
Intestinal FGF15 expression increases and prevents muscle mass loss after VSG in mice. **a** *FGF15* RNA expression in duodenum, jejunum and ileum in control Sham and VSG mice fed 60% HFD. Intestinal mucosa was collected 15 min post mixed meal gavage (*n* = 6 Sham and 7 VSG). **b** Experimental timeline. **c** Body weight and **d** food intake before surgery. **e** Body weight after surgery. **f** Fat body mass and **g** lean body mass before and after surgery. **h** Cumulative food intake and **i** energy efficiency for weeks 1–8 after surgery. **j** Food intake, **k** fecal energy and **l** absorbed energy during week 11 after surgery. Animal number: Control Sham (*n* = 6), Control VSG (*n* = 8), FGF15^INT-KO^ Sham (*n* = 8), FGF15^INT-KO^ VSG (*n* = 5). Data are shown as means ± SEM. Panels **a**, **c**, and **d** were analyzed with Student’s two-tailed *t* test. Panels **e**–**l** were analyzed with two-way ANOVA with Tukey’s post hoc test. *p < 0.05.

Numerous reports have also shown that plasma FGF19 levels increase after weight-loss surgeries^22,32,33,38–40^. Due to the lack of commercially available and validated FGF15 assays, we were unable to measure circulating FGF15 levels after VSG in mice^41,42^. However, our data show that ileal FGF15 expression increases after VSG in mice (Fig. 1a). These data point to FGF15/19 as a potential target to mediate the effects of weight-loss surgery.

Global ablation of FGF15 in *FGF15−/−* mice resulted in impaired glucose tolerance^18,19^, but in our hands, these mice are surprisingly protected against diet-induced obesity^43^.FGF15 is highly expressed in the developing mouse brain^44–46^. Expression of FGF15 in the adult mouse becomes limited to the distal intestine and dorsal medial hypothalamus^14,15,47^. Thus, we speculate that the total body knockouts have impaired development of the CNS, which likely contributes to their reduced weight gain on a HFD. These issues make it difficult to use the total body knockout to determine critical aspects of FGF15 function in adult animals. Therefore, we generated a FGF15 flox/flox mouse to test specific hypotheses about the tissue-specific role of FGF15 in metabolism after VSG. The FGF15 flox/flox mice were built using CRISPR-Cas9 with LoxP sites flanking exon 2 of the FGF15 gene. We bred these mice to VilCreERT2 mice and administered tamoxifen (intraperitoneal, 3 doses/150 mg/kg; Fig. 1b) to FGF15^INT-KO^ (VilCreERT2; FGF15 flox/flox) and Controls (6 VilCreERT2, 4 FGF15 flox/flox, and 4 FGF15 flox/+ were combined as there were no differences in the phenotypes of these animals; Fig. 1b).

After the studies were completed, we validated exon 2 excision within ileal mucosa (where FGF15 is most highly expressed) in all mice (Supplementary Fig. 1). FGF15^INT-KO^ Sham and VSG mice had undetectable expression of ileal *FGF15*, with no significant alterations in ileal *FXR* expression, although there was a trend of decreased expression in FGF15^INT-KO^ Sham compared to Control Sham (Supplementary Fig. 1a, b). All mice received tamoxifen at the same time, and a week later were placed on 60% high-fat diet (HFD; Fig. 1a). Prior to surgery, Control and FGF15^INT-KO^ mice had similar body weight increase in response to HFD, without differences in food intake, fat and lean mass (Fig. 1c, d, f, g).

After 8 weeks of being on HFD, each mouse underwent either a Sham or VSG procedure and was returned to HFD four days after surgery. VSG removes ~80% of the stomach along the stomach curvature (Fig. 1a). As expected, Control mice receiving VSG had sustained weight loss compared to Control Sham mice, and that weight loss came mostly from loss of fat mass (Fig. 1e, f). Control VSG mice maintained their lean mass even after surgery, as we have shown before^48^. FGF15^INT-KO^ VSG mice also lost a significant amount of weight, but 9 weeks post-surgery their body weight was significantly lower compared to Control VSG (Fig. 1e). Although FGF15^INT-KO^ VSG mice lost fat mass, surprisingly they also lost a significant amount of lean mass and this loss was significantly greater than when compared to Control VSG and both Sham groups (Fig. 1f, g).

Cumulative food intake and the average energy efficiency (change in body weight divided by food intake) for the 8 weeks after surgery showed that the body weight loss observed in FGF15^INT-KO^ VSG mice was not a result of decreased food intake (Fig. 1h, i). This introduced the possibility that the loss of intestinal FGF15 leads to increased malabsorption following VSG. We measured the absorbed energy by bomb calorimetry 11 weeks after surgery. The average daily food intake was not different between the groups (Fig. 1j). However, the fecal energy output was reduced in FGF15^INT-KO^ VSG mice compared to Control VSG and FGF15^INT-KO^ Sham mice (Fig. 1k). Given that all mice were on the same diet, FGF15^INT-KO^ VSG mice had increased their absorption of calories from ingested food (Fig. 1l). Thus, the additional weight and lean mass loss in FGF15^INT-KO^ VSG was not secondary to nutrient malabsorption.

### Loss in muscle mass is accompanied by decreased strength and skeletal muscle fiber size in FGF15^INT-KO^ VSG mice

Previous data has shown that exogenous administration or genetic overexpression of FGF19 decreases body weight and adiposity^20,24–28^, but prevents muscle mass wasting by enlarging muscle fiber size and protecting muscle from atrophy^49^. In addition to significant loss of muscle mass in FGF15^INT-KO^ VSG mice (Fig. 1g), we observed a trend of decreased grip strength (9 weeks post-surgery) and soleus muscle fiber size in these mice (Supplementary Fig. 2a, b). Moreover, a distribution analysis of the fiber size showed that FGF15^INT-KO^ VSG mice have a rightward shift towards an increased number of smaller and a lower number of larger soleus fibers (Supplementary Fig. 2c).

To further examine the atrophy-related pathways in soleus muscle, we measured circulating Myostatin (GDF8) and Activin A levels and the soleus muscle expression of their downstream targets, muscle-specific E3 ubiquitin ligases Atrogin 1 and MuRF1. We did not observe any differences in the plasma Myostatin and Activin A levels, nor the RNA expression of Atrogin 1 (encoded by *Fbxo32*) and MuRF1 (encoded by *Trim63*) in soleus muscle (Supplementary Fig. 2d–g). Next, we examined plasma levels of IGF1 and FGF21 as anti and pro-atrophy mediators in skeletal muscle. Twelve weeks after surgery postprandial plasma levels of IGF1 were decreased in FGF15^INT-KO^ VSG mice compared to Control VSG (Supplementary Fig. 2h). Studies have indicated that FGF21 levels increase with fasting and FGF21 is necessary for the fasting-induced muscle mass and force loss^50^. FGF15^INT-KO^ VSG mice had increased plasma levels of FGF21 compared to Control VSG and FGF15^INT-KO^ Sham mice (Supplementary Fig. 2i). In addition, there was a significant negative correlation between muscle mass and plasma FGF21 levels (Supplementary Fig. 2j). Circulating amino acid levels 6-weeks post-surgery were not different among the groups (Supplementary Fig. 2k).

FGF15^INT-KO^ VSG mice did not show differences in small bowel biometry (Supplementary Fig. 3a–c). The large bowel weight was significantly less in FGF15^INT-KO^ VSG compared to FGF15^INT-KO^ Sham mice (Supplementary Fig. 3d). However, there was also a trend toward decreased large bowel weight in Control VSG compared to Control Sham mice, suggesting that decrease in large bowel weight could be related to decreased body weight of the VSG groups (Supplementary Fig. 3d)^51^. The ratio of large bowel weight/length was significantly reduced in both VSG groups compared to Control Sham but was not significantly different compared to FGF15^INT-KO^ Sham mice (Supplementary Fig. 3f). Further analysis of ileum cross-sections showed no differences in villi height, crypt depth or the ratio of villi height/crypt depth (Supplementary Fig. 3g–i).

### Intestinal-derived FGF15 partially preserves bone and bone marrow adipose tissue (BMAT) loss following VSG

**Fig. 2:**
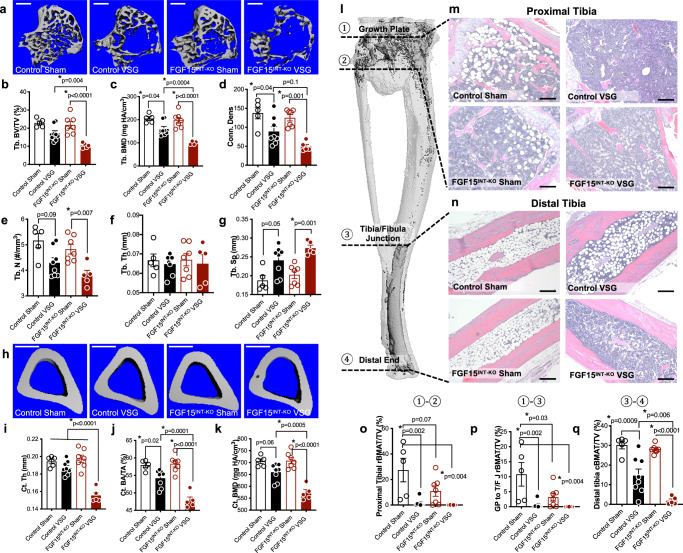
Intestinal-derived FGF15 partially preserves bone and bone marrow adipose tissue (BMAT) loss following VSG. **a** 3D images of trabecular bone, scale bars 500 μm. **b** Trabecular bone volume fraction (Tb. BV/TV), **c** trabecular bone mineral density (Tb. BMD), **d** trabecular bone connective density (Conn. Des) and **e** trabecular number (Tb. N), **f** thickness of the trabecular bone (Tb. Th), **g** spacing between trabeculae (Tb. Sp). **h** 3D images of mid-cortical bone, scale bars 500 μm. **i** Thickness of the cortical bone (Ct. Th). **j** Cortical bone area (Ct. BA/TA) and **k** cortical bone mineral density (Ct. BMD). **l** Tibial BMAT was visualized by osmium staining. **m**, **n** Representative sections from proximal and distal tibiae were stained with H&E and are shown at ×100 magnification, scale bars 200 μm. **l** Tibial BMAT was quantified relative to total bone volume after osmium staining within the indicated regions as shown in (**o**) proximal Tibia (①-②), **p** growth plate (G/P) to tibia/fibula junction (T/F J) (①-③), **q** distal tibia is T/F J to distal end (③-④). Animal number: Control Sham (*n* = 5), Control VSG (*n* = 8), FGF15^INT-KO^ Sham (*n* = 7), FGF15^INT-KO^ VSG (*n* = 5). Data are shown as means ± SEM. *p < 0.05 (two-way ANOVA with Tukey’s post hoc test).

Previous studies in our lab have shown that loss of bone and BMAT following VSG is independent of body weight and diet^52^. In the current study, VSG caused a precipitous loss of trabecular and cortical bone loss in FGF15^INT-KO^ mice. Specifically, FGF15^INT-KO^ VSG mice had decreased trabecular bone volume fraction (Tb. BV/TV), trabecular bone mineral density (Tb. BMD), and trabecular connective density (Conn. Dens) (Fig. 2a–d). Although the thickness of the trabecular bone (Tb. Th) was not altered, the trabecular number (Tb. N) was decreased, while the spacing between trabeculae was increased (Tb. Sp) in FGF15^INT-KO^ VSG mice (Fig. 2e–g). Cortical thickness (Ct. Th), bone area (Ct. BA/TA), and bone mineral density (Ct. BMD) were reduced by VSG and further decreased by lack of intestinal-derived FGF15 (Fig. 2h–k).

Consistent with our previous studies, both “regulated” bone marrow adipose tissue, BMAT (rBMAT) in proximal tibia and “constitutive” BMAT (cBMAT) in distal tibia were decreased by VSG (representative images Fig. 2m, n)^52,53^. Interestingly, loss of intestinal FGF15 caused a baseline reduction of rBMAT in proximal tibia ranging from growth plate (GP) to tibia/fibula junction (T/F J), with nearly complete depletion after VSG (Fig. 2m, o, p). Although the distal tibial cBMAT of Sham mice was unaffected by loss of intestinal FGF15, this BMAT depot was more thoroughly depleted in FGF15^INT-KO^ VSG mice compared to Control VSG mice (Fig. 2n, q).

### Loss of intestinal FGF15 increases circulating fibroblast growth factor 23 (FGF23) and erythropoietin (EPO) levels after VSG

**Fig. 3:**
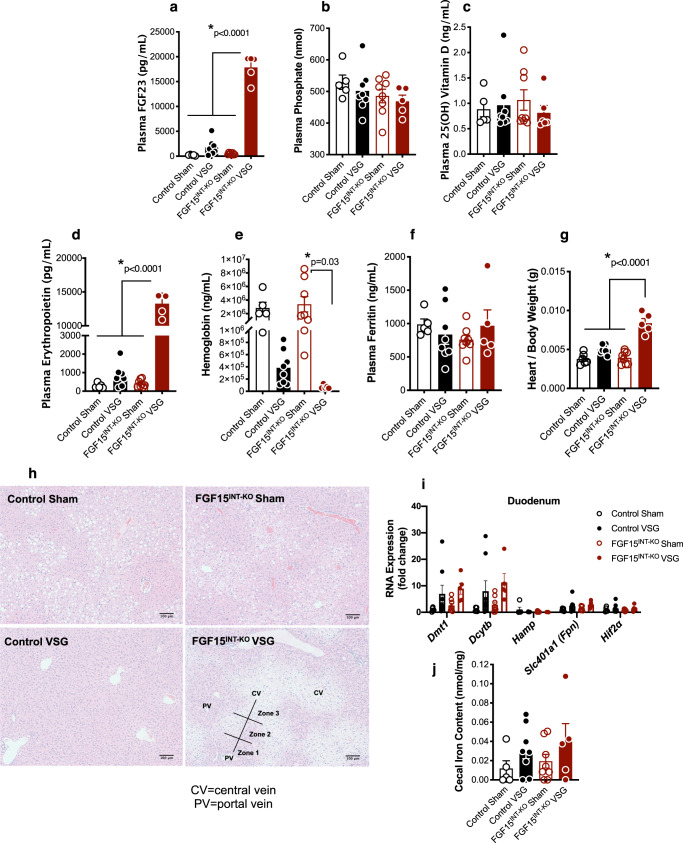
Loss of intestinal FGF15 increases circulating fibroblast growth factor 23 (FGF23) and erythropoietin (EPO) levels after VSG. Plasma levels of **a** FGF23, **b** phosphate, **c** 25(OH) vitamin D, **d** erythropoietin, **e** hemoglobin and **f** ferritin. **g** Heart to body weight ratio. **h** H&E stain of liver sections, CV = central vein, PV = portal vein, Zones 1–3, scale bars 100 μm. **i** RNA expression of *Dmt1, Dcytb*, *Hamp*, *Slc401a1* (Fpn) and *Hif2α* in duodenum. **j** Iron levels in cecal contents. Animal number for (**a**–**f**) and (**i**, **j**): Control Sham (*n* = 5), Control VSG (n = 8), FGF15^INT-KO^ Sham (*n* = 8), FGF15^INT-KO^ VSG (*n* = 5). Animal number for g: Control Sham (*n* = 6), Control VSG (*n* = 8), FGF15^INT-KO^ Sham (n = 8), FGF15^INT-KO^ VSG (*n* = 5). Data are shown as means ± SEM. *p < 0.05 (two-way ANOVA with Tukey’s post hoc test).

Fibroblast growth factor 23 (FGF23), the third member of the endocrine FGFs in addition to FGF15/19 and FGF21, is a bone-derived hormone that regulates mineral homeostasis and consequently bone mineral density^54^. Plasma FGF23 levels were elevated almost 20,000-fold in FGF15^INT-KO^ VSG mice (Fig. 3a). Plasma phosphate and 25 (OH) vitamin D levels were unaffected (Fig. 3b, c). To investigate the cause for increased plasma FGF23 levels, we measured circulating erythropoietin (EPO), a known regulator of FGF23 and bone loss^55,56^. EPO was also increased by 15,000-fold in FGF15^INT-KO^ VSG mice (Fig. 3d). Increased plasma FGF23 and EPO levels are often observed in anemia. Hemoglobin levels were decreased in FGF15^INT-KO^ VSG compared to FGF15^INT-KO^ Sham mice (Fig. 3e). Plasma ferritin levels were unaffected (Fig. 3f). Consistent with high levels of EPO, heart to body weight ratio (Fig. 3g) was increased in FGF15^INT-KO^ VSG mice. Moreover, H&E stains in liver sections showed zonal injury pattern (Fig. 3h). Zone 3 around the central hepatic veins appeared paler compared to zones 1 and 2 (Fig. 3h). These patterns are consistent with relative hypoxia, possibly secondary to anemia. The expression of hypoxia-inducible factor 2 alpha (*Hif2α*) and its target genes were upregulated in the duodenum following VSG and independent of genotype, specifically those genes transcribing the iron transporters divalent metal transporter Dmt1 (encoded by *Slc11a2*), duodenal cytochrome B Dcytb (encoded by *Cybrd1*), and Ferroportin (encoded by *Slc401a1*) (Fig. 3i). Duodenal expression of hepcidin (*Hamp*) was not altered by VSG or lack of gut-derived FGF15 expression (Fig. 3i). Lastly, iron content in cecal contents was not different between the groups but showed a trend of increased levels in both VSG groups (Fig. 3j).

### Loss of intestinal FGF15 increases circulating total GLP-1 levels and increases gastric emptying after VSG

**Fig. 4:**
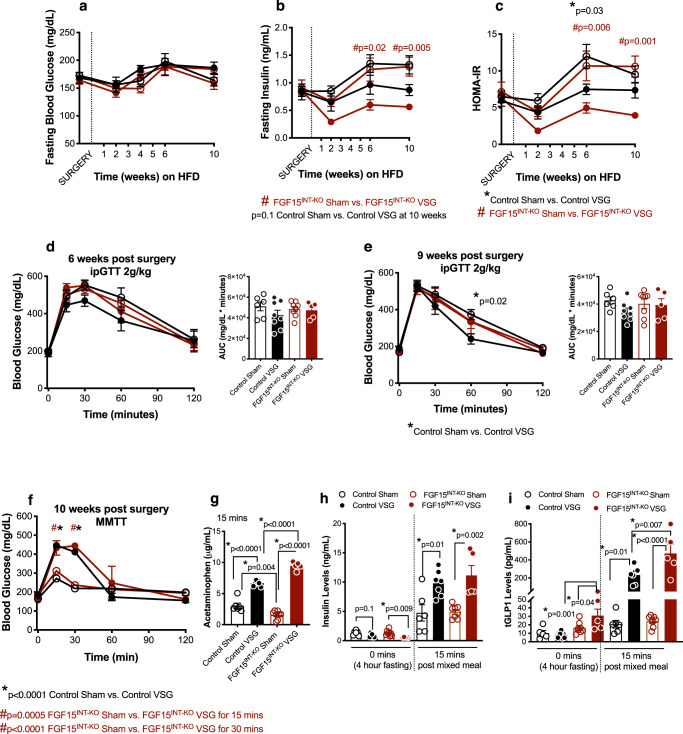
FGF15^INT-KO^ mice remain glucose intolerant despite significant body weight loss after VSG. **a** Fasting (4 h) blood glucose, **b** fasting (4 h) insulin levels and **c** HOMA-IR after surgery. **d** Intraperitoneal glucose tolerance test (ipGTT; 2 g/kg) performed 6-weeks post-surgery and Area Under the Curve (AUC). **e** Intraperitoneal glucose tolerance test (ipGTT; 2 g/kg) performed 9 weeks post-surgery and Area Under the Curve (AUC). **f** Mixed meal tolerance test (MMTT) performed 10 weeks post-surgery. **g** Gastric emptying rate measured by acetaminophen levels at 15 min post mixed meal. **h** Insulin levels at baseline (4 h fast) and 15 min post mixed meal. **i** Total GLP-1 levels at baseline (4 h fast) and 15 min post mixed meal. Animal number (**a**–**f**) and (**h**, **i**): Control Sham (*n* = 6), Control VSG (*n* = 8), FGF15^INT-KO^ Sham (*n* = 8), FGF15^INT-KO^ VSG (*n* = 5). Animal number g: Control Sham (*n* = 6), Control VSG (*n* = 7), FGF15^INT-KO^ Sham (*n* = 8), FGF15^INT-KO^ VSG (*n* = 5). Data are shown as means ± SEM. *p < 0.05 (two-way ANOVA with Tukey’s post hoc test).

Four-hour fasted blood glucose concentrations were not different among any of the four groups, but insulin concentrations and HOMA-IR were lower in FGF15^INT-KO^ VSG compared to FGF15^INT-KO^ Sham (Fig. 4a–c). As expected, we saw improvements in glucose tolerance in Control VSG compared to Control Sham mice six- and nine-weeks post-surgery (Fig. 4d, e). To our surprise, there was no difference in glucose tolerance between FGF15^INT-KO^ VSG and FGF15^INT-KO^ Sham mice (Fig. 4d, e).

Next, we challenged mice with a mixed meal for the assessment of postprandial glucose excursion, insulin, and GLP-1 response. Acetaminophen, which is rapidly absorbed once it leaves the stomach was added to the mixed meal to assess gastric emptying rate. FGF15^INT-KO^ Sham mice had a similar glucose excursion curve, but decreased gastric emptying rate compared to Control Sham mice (Fig. 4f, g). Both VSG groups responded to the mixed meal with similar glucose excursion curves (Fig. 4f), significantly higher than their Sham controls as a result of increased gastric emptying rate (Fig. 4g). Despite similar glucose excursion, FGF15^INT-KO^ VSG mice showed significantly higher gastric emptying rate (indicated by the greater plasma acetaminophen levels) compared to Control VSG mice (Fig. 4g). Basal and postprandial insulin levels were increased in both VSG groups compared to Sham controls (Fig. 4h). Interestingly, basal and postprandial total GLP-1 levels were significantly higher in FGF15^INT-KO^ VSG mice compared to all other groups (Fig. 4i).

### Loss of intestinal FGF15 results in aberrant hepatic lipid and glycogen metabolism following VSG

**Fig. 5:**
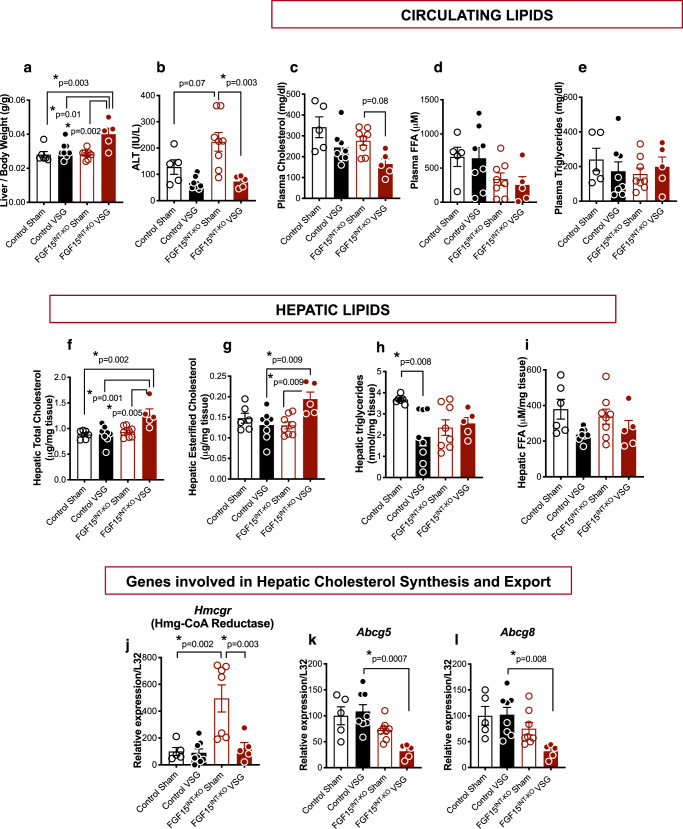
Loss of intestinal FGF15 results in aberrant hepatic lipid metabolism following VSG. **a** Liver weight normalized to body weight. **b** Alanine aminotransferase (ALT) plasma levels. **c** Plasma cholesterol. **d** Plasma-free fatty acids (FFA) and **e** Plasma triglycerides (postprandial, 12 weeks post-surgery). **f** Total hepatic cholesterol. **g** Hepatic esterified cholesterol. **h** Hepatic triglycerides. **i** Hepatic free fatty acids. Hepatic RNA expression of cholesterol synthesis gene **j**. *Hmcgr* and cholesterol export genes **k**. *Abcg5* and **l**. *Abcg8*. Animal number for (**a**–**i**) and (**k**, **l**): Control Sham (*n* = 5), Control VSG (*n* = 8), FGF15^INT-KO^ Sham (*n* = 8), FGF15^INT-KO^ VSG (*n* = 5). Animal number for j: Control Sham (*n* = 5), Control VSG (*n* = 8), FGF15^INT-KO^ Sham (*n* = 7), FGF15^INT-KO^ VSG (*n* = 5). Data are shown as means ± SEM. *p < 0.05 (two-way ANOVA with Tukey’s post hoc test).

FGF15^INT-KO^ VSG had increased liver to body weight ratio (Fig. 5a). Assessment of glycogen stores revealed that FGF15^INT-KO^ VSG have decreased liver glycogen and a trend toward higher skeletal muscle (tibialis anterior) glycogen content (Supplementary Fig. 4a, b). Postprandial plasma alanine aminotransferase (ALT) levels, a predictor of liver injury, and plasma cholesterol levels trended to be reduced in both VSG groups compared to Sham controls (Fig. 5b, c), without changes in plasma-free fatty acids or plasma triglycerides (Fig. 5d, e).

Analysis of hepatic lipids revealed an increased cholesterol and esterified cholesterol levels in FGF15^INT-KO^ VSG mice (Fig. 5f, g). Hepatic triacylglycerols were decreased in Control VSG compared to Control Sham mice, but remained at similar levels between FGF15^INT-KO^ Sham and FGF15^INT-KO^ VSG mice (Fig. 5h). There was a trend toward decreased hepatic free fatty acids in both VSG groups compared to Sham controls (Fig. 5i). Surprisingly, the cholesterol synthesis rate-limiting gene, 3-hydroxy-3-methyl-glutaryl-glutaryl-coenzyme A reductase, Hmg-CoA reductase (*Hmcgr*), was upregulated in FGF15^INT-KO^ Sham compared to Control Sham mice, but reduced after VSG in FGF15^INT-KO^ VSG mice (Fig. 5j). Next, we measured the expression in hepatic cholesterol efflux pump-ATP-binding cassette, sub-family G, members 5 and 8 (*Abcg5* and *Abcg8*) and found that their expression was decreased in FGF15^INT-KO^ VSG mice (Fig. 5k, l). These data suggest that despite decreased cholesterol synthesis, there is attenuated cholesterol export leading to elevated liver cholesterol content in FGF15^INT-KO^ VSG mice.

The hepatic expression of *FXR* was similar in Control and FGF15^INT-KO^ mice (Supplementary Fig. 5a). We did see a trend of decreased expression of *FXR* in both VSG groups compared to their respective Sham groups (Supplementary Fig. 5a). FXR target small heterodimer partner, *SHP*, was not significantly different, but showed a trend for decreased expression in both VSG groups. (Supplementary Fig. 5b). Fatty acid oxidation and lipid metabolism genes fatty acid synthase (*FAS*) and peroxisome proliferator-activated receptor alpha *(PPAR alpha)* were decreased in FGF15^INT-KO^ VSG mice compared to FGF15^INT-KO^ Sham controls (Supplementary Fig. 5c, d). Although not significant, there was a trend of decreased expression of these genes in Control VSG compared to Control Sham mice (Supplementary Fig. 5c, d). *PPAR alpha* target gene, Carnitine palmitoyltransferase 1A (*Cpt1a*) essential for converting long-chain fatty acids into energy (lipogenesis), showed a trend toward decreased expression after VSG in FGF15^INT-KO^ but not Control mice (Supplementary Fig. 5e). Interestingly, cluster of differentiation 36 (*CD36*), a cell surface protein that imports fatty acids, was increased in FGF15^INT-KO^ VSG compared to Control VSG mice (Supplementary Fig. 5f).

### Intestinal FGF15 regulates enterohepatic bile acid metabolism following VSG

**Fig. 6:**
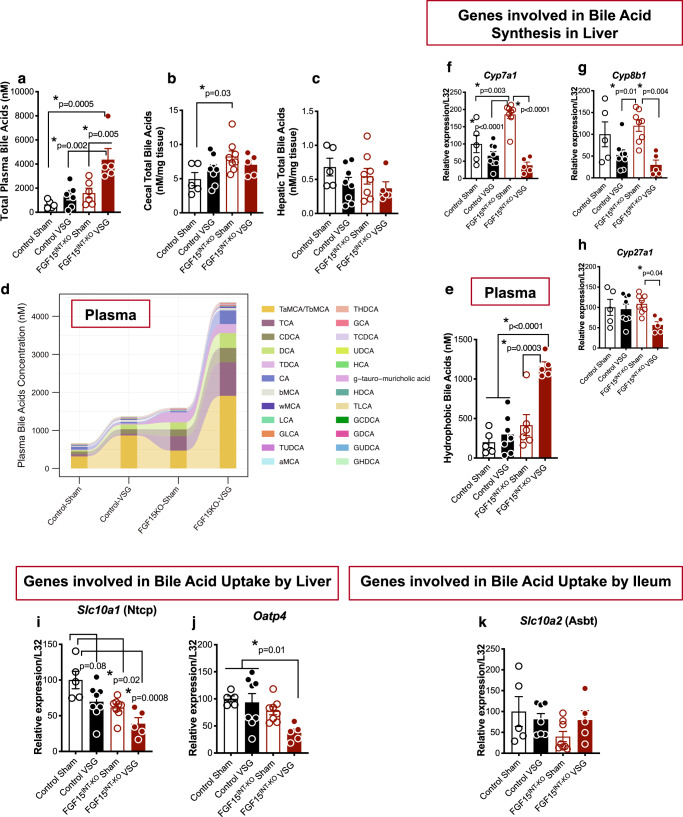
Intestinal FGF15 regulates enterohepatic bile acid metabolism following VSG. **a** Total plasma bile acid levels. **b** Cecal contents total bile acid levels. **c**. Hepatic total bile acids. **d** Plasma bile acid composition. **e** Hydrophobic plasma bile acid levels. Hepatic RNA expression of bile acid synthesis genes. **f** *Cyp7a1*, **g** *Cyp8b1* and **h** *Cyp27a1*. Hepatic RNA expression of bile acid uptake genes **i** *Slc10a1* (coding for Ntcp) and **j** *Oatp4*. **k** Ileum RNA expression of bile acid uptake gene *Slc10a2* (coding for Asbt). Animal number for (**b**), (**c**), (**f**–**j**): Control Sham (*n* = 5), Control VSG (*n* = 8), FGF15^INT-KO^ Sham (*n* = 8), FGF15^INT-KO^ VSG (*n* = 5). Animal number for (**a**), (**d**), (**e**): Control Sham (*n* = 5), Control VSG (*n* = 7), FGF15^INT-KO^ Sham (*n* = 6), FGF15^INT-KO^ VSG (*n* = 5). Animal number for k: Control Sham (n = 5), Control VSG (*n* = 7), FGF15^INT-KO^ Sham (*n* = 8), FGF15^INT-KO^ VSG (*n* = 5). Data are shown as means ± SEM. *p < 0.05 (two-way ANOVA with Tukey’s post hoc test).

FGF15 is expressed in ileal enterocytes of the small intestine and released postprandially in response to bile acid absorption. Consistent with this role to inhibit bile acid production, plasma and cecal content concentrations of bile acids are higher in mice lacking intestinal FGF15 (Fig. 6a, b). Moreover, plasma bile acid composition revealed higher levels of TCA (taurocholate), CDCA (chenodeoxycholate), DCA (Deoxycholate), TDCA (taurodeoxycholate), and TCDCA (Fig. 6d). VSG has been shown to alter both the concentration and composition of bile acids. Therefore, we assessed the role that FGF15 might play in the effect of VSG to alter bile acids. Control VSG mice had a trend of increased plasma bile acid levels, with higher levels of TαMCA (tauro-α-muricholate)/ TβMCA (tauro-β-muricholate), TCA, DCA, and TDCA levels (Fig. 6d, Supplementary Fig. 6). FGF15^INT-KO^ VSG mice had much higher plasma bile acid levels, but normal cecum content of bile acid levels when compared to Control VSG and FGF15^INT-KO^ Sham mice (Fig. 6a, b). FGF15^INT-KO^ VSG mice also had higher plasma TαMCA/TβMCA levels (similar to Control VSG), but also high levels of TCA, CDCA, DCA, TDCA, CA (cholate) (Fig. 6D, Supplementary Fig. 6). The levels of hydrophobic bile acids (GCA/glycocholate, CA/cholate, CDCA/chenodeoxycholate, DCA/deoxycholate, LCA/lithocholic acid) in plasma were increased by more than two-fold in FGF15^INT-KO^ VSG compared to Control VSG mice (Fig. 6e, Supplementary Fig. 6). Hepatic total bile acid levels were not different but showed a trend of decreased levels in both VSG groups (Fig. 6c). The ratios of cholesterol to bile acids in plasma, liver, and cecal content showed that lack of gut-derived FGF15 after VSG drives a system for increased bile acids in plasma and increased cholesterol in the liver (Supplementary Fig. 7a–c). Not surprisingly, lack of intestinal FGF15 resulted in higher expression of the bile acid synthesis gene *Cyp7a1* in FGF15^INT-KO^ Sham compared to Control Sham mice (Fig. 6f). However, despite the fact that FGF15^INT-KO^ VSG mice lack intestinal FGF15 and have increased plasma bile acid levels, their expression of hepatic bile acid synthesis genes cholesterol 7a-hydroxylase *(Cyp7a1)*, sterol 12-alpha-hydroxylase *(Cyp8b1),* and sterol 27-hydroxylase *(Cyp27a1)* were decreased (Fig. 6f–h).

Next, we measured the expression of hepatic and ileal bile acid uptake transporters. The expression of *Slc10a1* (coding for liver bile acid transporter Ntcp) and *Oatp4* was decreased in FGF15^INT-KO^ VSG mice (Fig. 6i, j). We did not see differences in the expression of bile acid transporter *Slc10a2* (coding for apical sodium-dependent bile acid transporter, Asbt) in the ileum (Fig. 6k). These data suggest that bile acid uptake by liver is reduced in FGF15^INT-KO^ VSG mice, potentially contributing to the increased plasma bile acid levels.

### Intestinal FGF15 modulates microbiota in cecal content

**Fig. 7:**
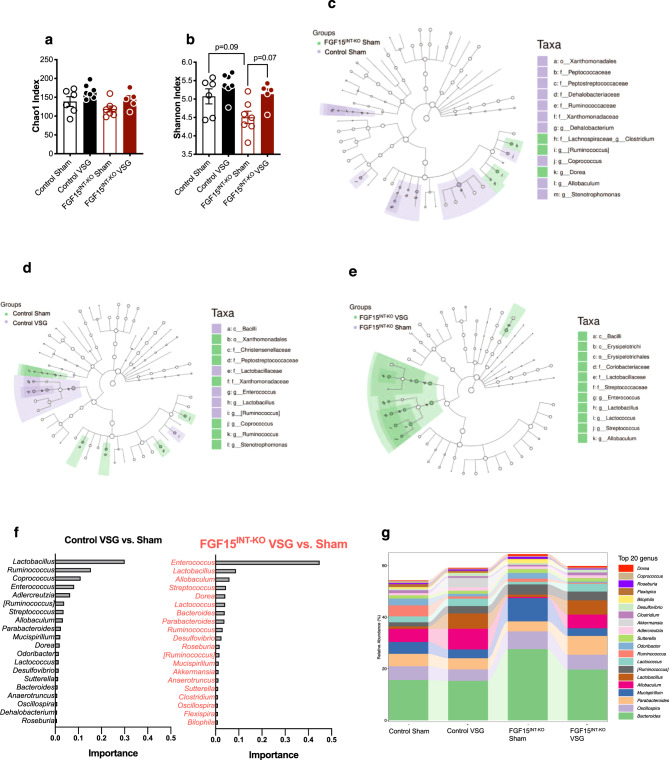
Intestinal FGF15 modulates microbiota in cecal content. **a** Chao1 abundance and **b** Shannon index diversity of the gut microbiota in the cecal contents. LEfSex analysis depicting nodes within the bacterial taxonomic hierarchy that are enriched in cecal microbiota from **c** Control Sham versus FGF15^INT-KO^ Sham and **d** Control Sham versus VSG and **e** FGF15^INT-KO^ Sham versus VSG. Diagrams generated by LEfSe indicating differences at *phylum*, *order*, *class*, *family*, and *genus* levels between the two groups. **f** Top-ranked taxa at *genus* level identified by random forest analysis according to their ability to discriminate the microbiota of Sham and VSG mice per genotype in decreasing order of discriminatory importance. A comparison of the abundance of markers in VSG relative to Sham counterparts in each genotype. **g**. Differences in relative abundance of taxa at *genus* level. Animal number Control Sham (*n* = 6), Control VSG (*n* = 7), FGF15^INT-KO^ Sham (*n* = 7), FGF15^INT-KO^ VSG (*n* = 5). Data in (**a**, **b**) are shown as means ± SEM. *p < 0.05 (two-way ANOVA with Tukey’s post hoc test).

Changes in gut microbiota composition are considered a potential contributor to the metabolic benefits of bariatric surgery^57–59^. We investigated whether intestinal FGF15 modulates shifts in the microbial communities by VSG. To determine the effects of FGF15 on VSG-induced gut microbiota alteration, we performed *16S* ribosomal RNA (rRNA) gene sequencing on cecal content samples collected 12 weeks after surgery (collected at the time of sacrifice). Chao1 and Shannon index were used to estimate with-in sample richness and diversity, respectively. The former represents the total number of microbes present in one single sample, while the latter accounts for both richness and evenness of the microbes. We did not see significant changes in richness (Chao1 index) and diversity (Shannon index) (Fig. 7a, b). However, there was a trend toward increased richness in both VSG groups compared to their respective Sham controls. In addition, we observed a trend of decreased cecal diversity in FGF15^INT-KO^ Sham compared to Control Sham mice (Fig. 7b).

Linear discriminant analysis (LDA) effect size (LEfSe) analysis showed taxonomic differences in the microbiota composition of the cecal content between Control Sham and FGF15^INT-KO^ Sham groups (Fig. 7c). *Firmicutes* and *Bacteroidetes* are the most abundant *phyla* in fecal microbiota. The relative proportion of *Firmicutes* and *Bacteroidetes* has been reported to be affected differently by obesity and high-fat feeding *Firmicutes* at the *phylum* level, *Erysipelotrichales* at *order* level, and *Allobaculum* and *Ruminococcus* at the *family* level showed a trend of decreased abundance in FGF15^INT-KO^ Sham compared to Control Sham (Supplementary Fig. 8b, e, h, i). However, *Lachnospiraceae* at the *family* level and *Bacteroides* at the *genus* level were increased in FGF15^INT-KO^ Sham compared to Control Sham (Supplementary Fig. 8f, g).

LDA effect size (LEfSe) analysis showed taxonomic differences in the microbiota composition of the cecal content between Sham and VSG in both genotype groups (Fig. 7d, e). To further define bacteria characteristic of enrichment of Sham and VSG in each genotype, we also performed a random forest model. *Lactobacillus* species have been suggested to confer beneficial effects in a broad spectrum^60,61^. At the *genus* level, *Lactobacillus*, with an importance score of 0.301 determined by the decrease in the classification accuracy when it was ignored, was identified as the top-ranked discriminator for the Control VSG compared to Control Sham gut microbiota. On the other hand, *Enterococcus* genus was identified as the top discriminator for FGF15^INT-KO^ VSG vs. FGF15^INT-KO^ Sham gut microbiota, with *Lactobacillus* genus having an importance score of 0.088. (Fig. 7f, g).

## Discussion

Increased circulating bile acids are one of the hallmarks of bariatric surgery, and our group and others have identified bile acid signaling through FXR as a molecular link mediating the beneficial metabolic effects of elevated bile acids following VSG^12,13^. Secretion of FGF15/19 is a key response to FXR activation. Consistent with human data that show increased circulating levels of FGF19 after bariatric surgery^22,32,33,38–40^, VSG in mice upregulates FGF15 expression in the ileum (Fig. 1). Here we demonstrate that intestinal FGF15 is necessary for many of the metabolic, morphologic, and microbiome responses to VSG.

To study the specific role of intestinal FGF15 in the response to VSG, we built a mouse model that allowed for specific deletion of FGF15 from the intestine in the adult animal (see Fig. 1 and “Methods”). Compared to control animals, these mice have similar body weight, body fat, food intake, and glucose regulation when challenged on a HFD. These data argue that intestinally derived FGF15 is not necessary for physiologic regulation of energy balance and glucose levels in diet-induced obese animals without bariatric surgery. It is important to note that these findings differ from gut-specific ablation of FXR, findings that were hypothesized to be dependent upon FXR’s downstream target FGF15^62,63^. Gut-specific FXR knock out mice (VilCre; FXR flox/flox) are metabolically fit and resistant to HFD-induced metabolic disease^63,64^. However, ablation of FXR in adult mice (VilCreERT2; FXR flox/flox) does not lead to resistance to HFD-induced obesity, similar to our FGF15^INT-KO^ Sham mice^13^. It is also important to note that we previously found that unlike wild-type mice, *FXR−/−* mice did not maintain body weight loss and did not have improved glucose tolerance after VSG and while maintained on HFD^12^. A caveat of these studies is that *FXR−/−* mice are resistant to HFD-induced obesity and glucose intolerance, and therefore, the window for improvement in these parameters is smaller^65^. This is also the case for total body *FGF15−/−* mice, as we also recently reported^43^. The key point is that it is likely that FXR and FGF15’s role in metabolic regulation is different in various tissues and it is also sensitive to ablation during development versus in the adult mouse. These are critical components for interpreting the role bile acid metabolism plays following bariatric surgery and a major rationale for why we built a model that would allow for tissue-specific deletion of FGF15 in the adult mouse.

Once released from the ileum, FGF15 enters the portal venous circulation and travels to the liver where FGF15 binds to its receptor FGFR4 and represses de novo bile acid synthesis through suppression of the rate-limiting enzyme cholesterol 7a-hydroxylase (*Cyp7a1*)^14^. Therefore, bile acids and FGF15/19 inhibit further bile acid synthesis and facilitate communication between the small intestine and liver. As predicted, the lack of FGF15 resulted in increased plasma bile acids and cecal bile acid content in FGF15^INT-KO^ Sham mice (Fig. 6). After VSG, plasma bile acids were further increased in FGF15^INT-KO^ VSG mice, but cecal bile acids levels were suppressed (Fig. 6). This suggests that VSG directly alters the compartmental-specific bile acid pool independently of FGF15.

Patients with NAFLD have increased hepatic *Cyp7a1* levels^22^. Exogenous administration of FGF19 and its analog NGM282 did not correct hyperglycemia in diabetic patients, but caused a rapid and sustained reduction in hepatic *Cyp7a1* levels and liver fat content in NAFLD patients^22^. Consistent with these observations, we saw increased hepatic expression of *Cyp7a1* in FGF15^INT-KO^ Sham mice compared to Control Sham (Fig. 6). However, hepatic *Cyp7a1* was significantly downregulated in FGF15^INT-KO^ VSG (Fig. 6). We speculate that the drastic increase in plasma bile acid levels in FGF15^INT-KO^ VSG mice downregulates hepatic *Cyp7a1* expression as a negative feedback independent of circulating FGF15^66,67^. The reduction of hepatic *Cyp7a1* in FGF15^INT-KO^ VSG mice is VSG-dependent and FGF15-independent (unlike observed in FGF15^INT-KO^ Sham). As there are higher levels of cholesterol, bile acid synthesis is facilitated because there is more substrate. However, *Cy7a1* is high in the FGF15^INT-KO^ Sham, because in the absence of FGF15 there is less to inhibit *de novo* synthesis. But, for FGF15^INT-KO^ VSG, the high amount of hepatic cholesterol and decreased hepatic bile acid uptake (decreased expression of *Slc10a1* (coding for Ntcp and *Oatp4*), leads to high plasma bile acids, creating negative feedback, and as a result *Cyp7a1* is downregulated in the FGF15^INT-KO^ VSG. Decreased hepatic *Slc10a1* and *Oatp4* and normal hepatic bile acid levels suggest that the liver is actively reducing bile acid uptake, leaving increased plasma bile acid pool. FGF15^INT-KO^ VSG mice have very high bile acids levels in plasma, but not in feces. This is consistent with FGF15^INT-KO^ VSG mice having increased recycling of bile acids compared to FGF15^INT-KO^ Sham. Ileal expression of *Slc10a2* (coding for Asbt) trended higher in FGF15^INT-KO^ VSG compared to FGF15^INT-KO^ Sham, which would lead to better reabsorption in the ileum, and decreased fecal bile acids. We speculate that if the bile acid recycling is higher in FGF15^INT-KO^ VSG, then there is no need to produce more bile acids, and therefore hepatic *Cyp7a1* expression decreases.

Further understanding of the mechanisms that suppress *Cyp7a1* would also be useful for cancer treatments that aim to block FGFR4^68^. FGF15/19 signaling has been implicated in the development of hepatocellular carcinoma, making FGFR4 antagonists attractive candidates for treating this disease^68^. However, blockade of FGFR4 signaling inhibits the FGF15/19-induced bile acid brake. The resulting elevation in hydrophobic bile acids, as seen in FGF15^INT-KO^ VSG mice, act as detergents and disrupt cell membranes, and can lead to liver damage^69^. Although we see significantly elevated levels of plasma bile acids and very high levels of hydrophobic bile acids (Fig. 6 and Supplementary Fig. 6) in the FGF15^INT-KO^ VSG compared to Control VSG mice, we do not observe signs of liver damage as noted by normal circulating levels of ALT in FGF15^INT-KO^ VSG mice (Fig. 5). Our data also found that intestinal FGF15 is necessary for the reduction in hepatic cholesterol content after VSG. FGF15^INT-KO^ VSG mice had increased liver weight (normalized to body weight), despite decreased hepatic glycogen content (Fig. 5 and Supplementary Fig. 4). Although plasma cholesterol levels were decreased in both groups after VSG, FGF15^INT-KO^ VSG mice had increased hepatic total and esterified cholesterol levels (Fig. 5). The cholesterol synthesis rate-limiting gene, Hmg-CoA reductase (*Hmcgr*), was upregulated in FGF15^INT-KO^ Sham compared to Control Sham mice, but surprisingly reduced after VSG in FGF15^INT-KO^ VSG mice (Fig. 5). Hepatic cholesterol efflux pump-ATP-binding cassette, sub-family G, members 5 and 8 (*Abcg5* and *Abcg8*) expression was decreased in FGF15^INT-KO^ VSG mice (Fig. 5). These data suggest that despite decreased cholesterol synthesis there is attenuated cholesterol export leading to elevated liver cholesterol content in FGF15^INT-KO^ VSG mice.

FGF21 levels were increased in FGF15^INT-KO^ mice (especially after VSG; Supplementary Fig. 2). FGF21 levels are tighly linked to nutrional status, and it has been well established that FGF21 levels increase in fasting and after ketogenic and low-protein diets^70–72^. The lack of FGF15, which is a postprandial hormone, could be altering the gut-liver communication and leading to the liver sensing a “fasting state” and increasing FGF21 levels^73^. Clinical and mouse studies have reported increased plasma FGF21 levels in patients and animal models of NAFLD^74–76^. Consistent with these data, we hypothesize that plasma FGF21 is increased in FGF15^INT-KO^ mice as compensatory mechanism to attenuate liver injury and hepatic lipid accumulation^77^. Importantly, we did not observe increased hepatic cholesterol levels in FGF15^INT-KO^ Sham mice compared to Control Sham (despite increased hepatic expression of *Hmcgr*). It is possible that the hepatic cholesterol in these mice is directed towards their increased plasma bile acid levels. It is also possible that increased FGF21 levels in FGF15^INT-KO^ Sham were sufficient to counteract the hepatic lipid accumulation and liver damage. Alternatively, other tissues, including muscle and adipose tissue, could be responsible for the increased FGF21 levels in FGF15^INT-KO^ VSG mice^78^. This is consistent with the observation that elevated plasma FGF21 levels have been linked to decreased muscle mass^50^ and reduced bone mineral density and BMAT^79–81^. Taken together these observations point to the possibility that plasma FGF21 levels are not a mediator of the profound cachexia observed in FGF15^INT-KO^ VSG mice and are increased in response to the muscle mass loss and/or hepatic lipid accumulation in these mice.

We did not find a difference in glucose excursion between Control Sham and FGF15^INT-KO^ Sham mice, suggesting that intestinal-derived FGF15 does not play an essential role in peripheral glucose tolerance, at least under HFD conditions (Fig. 4). As expected, we saw an improvement in glucose tolerance in Control VSG compared to Control Sham mice after intraperitoneal glucose challenge (ipGTT; Fig. 4). To our surprise, there was no difference in glucose tolerance between FGF15^INT-KO^ VSG and FGF15^INT-KO^ Sham mice, despite the large body weight loss in FGF15^INT-KO^ after VSG (Fig. 4). Loss in muscle mass as seen in FGF15^INT-KO^ VSG can greatly inhibit the proper disposal of glucose, leading to glucose intolerance. However, VSG in total body *FGF15−/−* mice also did not improve glucose tolerance despite lack of change in lean tissue mass, suggesting that these effects are FGF15-dependent and body weight-independent^43^. In addition, gallbladder bile diversion to the ileum, a surgical procedure that also results in sustained weight loss and improved glucose tolerance, is ineffective in restoring glucose tolerance in gut-specific ablation of FXR in adult mice^13^. Taken together, these data support the conclusion that the lack of improvement in glucose tolerance in our FGF15^INT-KO^ mice is dependent upon the enterohepatic system. Recent studies reported that patients who experience post-bariatric postprandial hypoglycemia have increased postprandial levels of FGF19, linking the levels of FGF15/19 to glucose regulation after weight-loss surgery^38^.

It has been reported that reduced expression of FGF15 in mice is associated with increased gastrointestinal motility and increased luminal water content, similar to human bile acid diarrhea^82^. However, gastric emptying was lower in FGF15^INT-KO^ Sham compared to Control Sham mice (Fig. 4). We speculate that the decrease in gastric emptying is a result of increased circulating bile acids in FGF15^INT-KO^ Sham mice, especially increased TDCA or DCA levels^83^. These differences in gastric emptying rates change after VSG. FGF15^INT-KO^ VSG had increased gastric emptying compared to Control VSG mice (Fig. 4). Consistent with previous reports, we also saw an increased total GLP-1 postprandial response in Control VSG mice after a mixed meal. The increase in total GLP-1 after VSG has been attributed to the rapid entry of ingested glucose and nutrients into the small intestine^84^. However, the GLP-1 (basal and postprandial) in FGF15^INT-KO^ VSG mice were much higher than Control VSG (Fig. 4). This points to a more complicated regulation of GLP-1 secretion beyond simply nutrient presentation. Strong evidence links bile acid activation of TGR5 to increased GLP-1 secretion^85–87^. Consequently, elevated levels of plasma bile acids, such as TLCA (TGR5 agonist), in FGF15^INT-KO^ mice given VSG may act to increase TGR5 signaling and drive increased GLP-1 secretion^88^. Interestingly, despite the elevated GLP-1 levels, FGF15^INT-KO^ mice given VSG do not have improved glucose tolerance pointing to a potentially more critical role of FGF15 as compared to GLP-1.

One of the most profound and surprising findings was that FGF15^INT-KO^ mice lose considerably more weight than the control mice after VSG (Fig. 1). While we have consistently observed that mice given a VSG lose little or no lean mass^48,89^, FGF15^INT-KO^ lose 25% of their lean tissue, much more than the control VSG mice (Fig. 1). Thus, while FGF15 does not mediate reductions in body fat after VSG, it is essential to protect from deleterious loss of lean mass after VSG. Loss of muscle mass is a major concern in the setting of rapid weight loss interventions such as very-low-calorie diets and bariatric surgery^90–93^. A second component of cachexia in the FGF15^INT-KO^ VSG mice was due to reduced bone mass and BMAT (Fig. 2). Bariatric procedures including VSG can result in bone loss beyond the normal response to reduced loading from weight loss, or calcium deficiency from diminished absorption^52,90,94^. In FGF15^INT-KO^ mice this effect of surgery is amplified and results in a diffuse pattern of osseous abnormality including both trabecular and cortical bone mass and BMAT (Fig. 2). In humans, reduction in bone density after bariatric surgery can predispose to fractures and is an important downside to the use of these procedures^90^. It remains to be seen whether this post-surgical metabolic bone disease can be accounted for by FGF15/19 signaling as suggested by our findings.

The cachexia observed in FGF15^INT-KO^ mice after VSG was surprising and we sought to identify potential mechanisms for the dramatic loss in muscle and bone mass in these mice. Fibroblast growth factor 23 (FGF23), the third member of the endocrine FGFs (in addition to FGF15/19 and FGF21), is a bone-derived hormone that regulates mineral homeostasis and consequently bone mineral density^54,56^. Plasma FGF23 levels were dramatically elevated in FGF15^INT-KO^ VSG mice (Fig. 3). Next, to investigate the source of increased plasma FGF23 levels, we measured circulating erythropoietin (EPO), a known driver for increased FGF23 and bone loss^55,56^. Further, elevated EPO is also sufficient to stimulate loss of bone and BMAT^95^. EPO was also highly increased in FGF15^INT-KO^ VSG mice (Fig. 3). Plasma phosphate and 25 (OH) vitamin D levels were unaffected, as has been reported in mice overexpressing EPO and high plasma FGF23 levels despite bone loss^54^. We were intrigued by these findings as increased plasma FGF23 and EPO levels are often observed in anemia. We have previously reported lower iron levels are observed in bariatric surgeries with widely varying impacts on duodenal exposure to chime, including VSG^96^. Hemoglobin levels were decreased in FGF15^INT-KO^ VSG compared to FGF15^INT-KO^ Sham mice, but ferritin levels were unaffected (Fig. 3). Both VSG mice trended toward increased iron levels in cecal contents but these were not FGF15-dependent. An increase in circulating EPO but normal ferritin levels and near compete depletion of BMAT, suggest that FGF15^INT-KO^ VSG mice have bone marrow dysfunction leading to anemia of chronic disease. Our previous studies have shown that loss of bone mass and BMAT after VSG is inversely correlated with myeloid cell expansion in marrow^52^. Therefore, the mechanism for these unexpected findings has to be a factor dependent on both FGF15 and VSG.

**Fig. 8:**
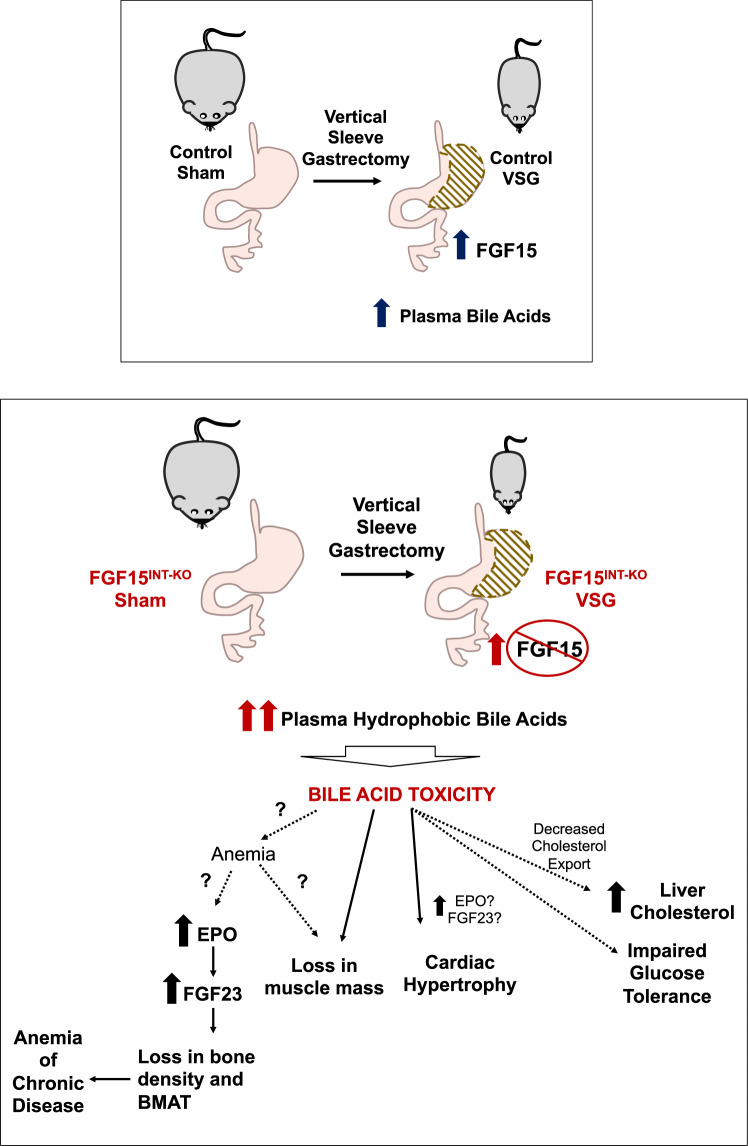
Intestinal-derived FGF15 protects against deleterious effects following sleeve gastrectomy in mice. VSG leads to an increase in ileal expression of *FGF15* and total plasma bile acids. In the absence of intestinally derived FGF15 (FGF15^INT-KO^), VSG leads to an even greater increase in circulating total and specifically, hydrophobic bile acids. We speculate that the increase in hydrophobic bile acids leads to bone marrow dysfunction, which increases EPO and FGF23 levels that likely contribute to potentially serious loss of bone mass, bone marrow adipose tissue (BMAT), inducing anemia of chronic disease. FGF15^INT-KO^ VSG mice have a loss of muscle mass and cardiac hypertrophy, as a result of bile acid toxicity or secondary to anemia of chronic disease. FGF15^INT-KO^ VSG mice also have increased hepatic cholesterol and they do not show improved glucose tolerance following VSG.

FGF15^INT-KO^ VSG mice had high levels of circulating hydrophobic bile acid levels (Fig. 6E, Supplementary Fig. 6). Hydrophobicity is an important factor for bile acid toxicity and cholestatic injury^97,98^. Therefore, we hypothesize that increased hydrophobic bile acids are an important contributor to the cachexia seen after VSG in FGF15^INT-KO^ mice (Fig. 8). The lack of gut-derived FGF15 is sufficient to increase circulating total bile acids (as can VSG), however, the combination of lack in gut FGF15 along with VSG results in very high levels of circulating total and most importantly, hydrophobic levels of bile acids (Fig. 6). Increase in circulating hydrophobic bile acids can lead to bile acid toxicity that may contribute to the cachexia observed in FGF15^INT-KO^ VSG mice. Further, VSG in total body *FGF15−/−* mice did not lead to cachexia consistent with relatively normal plasma levels of bile acids (compared to WT VSG)^43^.

Together, these data lead us to speculate that bile acids and bile acid toxicity is a critical contributor to the muscle and bone loss in FGF15^INT-KO^ VSG mice (Fig. 8). Recent study showed that antibiotic treatment and the resultant change in microbiota and intestinal bile acids results in skeletal muscle atrophy by decreasing bile acid-FXR-FGF15 signaling^99^. It has also been reported that exogenous FGF19 induces skeletal muscle hypertrophy and blocks muscle atrophy induced by glucocorticoid treatment, sarcopenia and obesity^49^. The mechanism behind these findings is not well understood, but bile acids may play a role. We also hypothesize that dramatic increases in EPO and FGF23 play a major role in bone density and BMAT loss in FGF15^INT-KO^ VSG mice. It remains unclear if and how bile acid toxicity induces anemia of chronic disease leading to the increase in EPO and FGF23. Further supporting the bile acid toxicity hypothesis, FGF15^INT-KO^ VSG mice have cardiac hypertrophy (Fig. 3). Elevated plasma bile acids that occur in mice with knock outs of both *FXR* and *Shp* or by pharmacological administration of the bile acids TCA and LCA also result in cardiac hypertrophy and reduced cardiac output^100,101^. In addition, anemia and increased FGF23 and EPO can also induce cardiac hypertrophy^102,103^. Future studies will dissect the effect of bile acid toxicity on the cardiovascular function of FGF15^INT-KO^ VSG mice, as these are important parameters for patients undergoing bariatric surgery procedures.

The critical point from these studies is that VSG increases circulating levels of bile acids by a mechanism that remains unclear. Our data lead us to hypothesize that the increase in FGF15 after VSG serves as an important break to limit this increase so as not to become deleterious and lead to potentially toxic levels of bile acids. In the absence of intestinally derived FGF15, VSG leads to an even greater increase in hydrophobic bile acids which we speculate leads to anemia of chronic disease and increased EPO and FGF23 levels that likely contribute to great loss of bone mass, BMAT, muscle mass, and cardiac hypertrophy. Circulating levels of FGF19 increase in humans after VSG, but this increase is variable between patients^22,32,33,38–40^. Our data suggest that bariatric surgery patients with low FGF19 levels may be at a higher risk for bone and skeletal muscle loss as well as increased hepatic cholesterol accumulation.

As with many studies using the mouse to model human effects, there are important caveats to the extrapolation to humans. Not all aspects of the regulation of enterohepatic circulation are identical in mouse and human and thus further work will be needed. These experiments are time and labor intensive so we have not directly tested whether lowering of plasma bile acids would prevent some deleterious effects of VSG in FGF15^INT-KO^ mice. Future work will directly address potential therapeutic strategies that could limit these deleterious effects of VSG. More research is warranted to determine if circulating FGF19 can act as a biomarker to identify patients that are at high risk for excessive bile acid levels and these negative outcomes following bariatric surgery. Understanding this relationship would allow physicians to optimize treatment strategies for at risk patients.

## Methods

### Study approval

All protocols complied with all relevant ethical regulations for animal testing and research. All protocols were approved by the University of Michigan (Ann Arbor, MI) Animal Care and Use Committees and were in accordance to NIH guidelines.

### Generation of FGF15 flox mouse

Gene sequence for the mouse FGF15 gene (4.4k base pair double strands DNA containing fgf15 exon) was downloaded from genebank. Guide RNAs were designed against the mouse sequence containing the region of interest for targeting (introns flanking FGF15 exon 2) using prediction algorithms available through http://crispor.tefor.net/. Guides were selected based on the specificity score and predicted efficiency (Mor.Mateos). Selected single guide RNA (sgRNA) sequences were subcloned into plasmid pX330 and subsequently confirmed by sequencing. Targeting DNA oligonucleotides including the 34 base pairs loxP site and 81-82 nucleotides of flanking sequence on either side were generated (IDT Technologies). DNA oligonucleotides were designed to disrupt the sgRNA and PAM sequences so that Cas9 would not be able to cleave the inserted sequence after incorporation into the genome. To test the ability of the sgRNAs to cut chromosomal DNA appropriately, each sgRNA was injected into fertilized eggs by the University of Michigan Transgenic Animal Core; zygotes were then allowed to develop into blastocysts in culture. PCR amplification of the target region followed by sequencing was used to confirm Cas9 dependent DNA cutting in vivo. Cas9/sgRNA/oligo donor were then injected into 300 fertilized mouse eggs (C57BL/6x SJL) and transferred to pseudo-pregnant recipients for gestation. After delivery of potential founders, tail DNA was isolated and screened by PCR across the region of interest to identify genomic manipulations. As founders are often mosaic for allelic changes, PCR products of genomic DNA were subcloned into topo vector and sequenced to characterize the genetic modification in the mice.

### Animals and diet

The FGF15 flox/flox mice, were built using CRISPR-Cas9 technology with LoxP sites flanking exon 2 of the FGF15 gene (described above). We bred these mice to VilCreERT2 mice (C57BL background) and administered tamoxifen (intraperitoneal, 3 doses/150 mg/kg) to VilCreERT2; Fgf15^flox/flox^ and controls (VilCreERT2 and Fgf15 flox/flox). We validated exon 2 excision within the latter jejunal and ileal mucosa, where FGF15 is most highly expressed (Supplementary Fig. 1). Male mice (6–8 weeks of age) were single-housed under a 12-h light/dark cycle with ad libitum access to water and food. A week after tamoxifen administration, FGF15^INT-KO^ and Control male mice (all littermates) were placed on 60% HFD from Research Diets, Inc. (New Jersey, US; Catalog D12492) for 8 weeks. Mice were singly-housed under a 12-h light/dark cycle in a facility maintained at 25 °C with 50–60% humidity for the duration of the studies. Mice underwent VSG or Sham surgery (described below) and returned to 60% HFD four days after surgery until the end of the study.

Animals were euthanized 12 weeks post-surgery (26-28 weeks of age). One Control Sham mouse died accidentally during NMR measurements on the day before necropsy. The post-necropsy data on metabolites and tissue gene expression for this Control Sham mouse was excluded, but the data not sensitive to nutritional state was included. The rest of the mice were fasted overnight, administered oral mixed meal (volume 200 µl Ensure Plus spiked with a 40-mg dextrose), and sacrificed 90 min later. Plasma and tissues were collected and frozen immediately. All animals were euthanized using CO_2._

### VSG in mice

Mice were maintained on a 60% HFD for 8 weeks prior to undergoing Sham or VSG surgery^48,104,105^. Mice were fasted overnight prior to day of surgery. Animals were anesthetized using isoflurane, and a small laparotomy incision was made in the abdominal wall. The lateral 80% of the stomach along the greater curvature and fundus was excised in VSG animals by using an Echelon Flex^TM^ powered vascular stapler (Ethicon Endo-Surgery, USA). The Sham surgery was performed by the application of gentle pressure on the stomach with blunt forceps for 15 seconds. All mice received one dose of Buprinex (0.1 mg/kg) and Meloxicam (0.5 mg/kg) immediately after surgery. All mice received Meloxicam (0.5 mg/kg) for 3 days after surgery and Enrofloxacin (40 mg/kg) for 5 days after surgery. Animals were placed on DietGel Boost (ClearH_2_O; Postland ME) for 3 days after surgery. They were placed back on pre-operative solid diet (60% HFD) on day 4 post-surgery. Body weight and food intake as well as overall health were monitored daily for the first 7 days after surgery and once weekly until end of the studies.

### Metabolic studies

Body weight was monitored monthly for 9 weeks prior and 12 weeks after Sham/VSG surgery. Intraperitoneal glucose tolerance test (IPGTT) was performed by intraperitoneal (IP) injection of 50% dextrose (2 g/kg) in 4-h fasted mice. Mixed-meal tolerance test (MMTT) was performed via an oral gavage of liquid meal (volume 200 µl Ensure Plus spiked with a 40-mg dextrose and 4-mg acetaminophen, Sigma-Aldrich) in 4-hour fasted mice. Blood was obtained from the tail vein and blood glucose was measured with Accu-Chek blood glucose meter (Accu-Chek Aviva Plus, Roche Diagnostics). Blood was collected from the tail vein at baseline and 15 min after gavage in EDTA-coated microtubes. Plasma acetaminophen levels were used to assess the rate of gastric emptying and were measured using spectrophotometry assay (Sekisui Diagnostics).

### ELISA and metabolite assays

Insulin (Crystal Chem), total GLP-1 (MesoScale Discovery), and acetaminophen (Sekisui Diagnostics) were measured during experiments shown in Fig. 3. Insufficient amount of blood was obtained from one Control VSG for insulin, total GLP-1 and acetaminophen levels at 15 min. Postprandial plasma obtained at termination of studies (see above for details) was used to measure IGF-1 (R&D Systems), Activin-A (R&D Systems), Myostatin (GDF8) (R&D Systems), FGF21 (R&D Systems), L-amino acids (Abcam), Erythropoietin (Abcam), FGF23 (Abcam), Ferritin (Abcam), Hemoglobin (Abcam), Phosphate (Abcam), 25(OH) Vitamin D (Abcam). Iron levels in cecal contents were measured with Iron Assay Kit (Abcam). Glycogen content was measured in liver and muscle (tibialis anterior) samples with Glycogen Assay kit (Sigma-Aldrich). All sampled blood was collected via tail vein in EDTA-coated tubes. All assays were performed according to the manufacturer’s instructions.

### Plasma bile acid composition

Plasma bile acid composition was measured by the University of Michigan Metabolomics Core using two-step solvent extraction. Supernatants are combined, dried, and re-suspended for LCMS separation by RPLC and measurements by ESI^-^ QQQ MRM methods^106^. *Sample preparation*: 20 µL plasma was transferred to a microtube. Eighty microliters of chilled acetonitrile with 5% NH_4_OH, containing isotope-labeled internal standards was added to the tube, and the mixture vortexed until completely homogenized. The mixture was incubated on ice for 10 min and vortexed to remix. This homogenized mixture was centrifuged and 3 µL of the supernatant from each sample was removed to create a pooled sample for QC purposes. Next, 90 µL of the supernatant transferred to an LC-MS autosampler insert and brought to dryness in a speedvac set to 45 °C for ~45 min. Each sample was reconstituted in 100 µL of 50/50 Methanol/Water. A series of calibration standards ranging from 0 to 1000 nM were prepared along with samples to quantify metabolites. *LC-MS analysis*: LC-MS analysis was performed on an Agilent system consisting of a 1290 UPLC module coupled with a 6490 Triple Quad (QqQ) mass spectrometer (Agilent Technologies, Santa Clara, CA) operated in MRM mode. MRM transitions are included in the table below. Metabolites were separated on a 100 mm × 2.1 mm Acquity BEH UPLC (1.7 µm) column (Waters Corp, Milford, MA) using H_2_O, 0.1% Formic acid, as mobile phase A, and Acetonitrile, 0.1% Formic acid, as mobile phase B. The flow rate was 0.25 mL/min with the following gradient: linear from 5 to 25% B over 2 min, linear from 25 to 40% B over 14 min, linear from 40 to 95% B over 2 min, followed by isocratic elution at 95% B for 5 min. The system was returned to starting conditions (5% B) in 0.1 min and held there for 3 min to allow for column re-equilibration before injecting another sample. The mass spectrometer was operated in ESI- mode according to previously published conditions. Data were processed using MassHunter Quantitative analysis version B.07.00. Metabolites were normalized to the nearest isotope-labeled internal standard and quantitated using two replicated injections of five standards to create a linear calibration curve with accuracy better than 80% for each standard. Using ROUT method and treating all the values in all subgroups as one set of data (*Q* = 1), we identified three samples (one Control VSG and two FGF15^INT-KO^ Sham) as significant outliers. QQ plot and Homoscedasticity plot showed that these samples were out of the normal distribution. In addition, analysis of total bile acids in the same samples using Total Bile Assay (NBT Method) (GenWay Biotech Inc; San Diego, CA) also revealed that these samples are abnormally elevated. Therefore, these samples were excluded from all plasma bile acid data analysis. Plasma bile acid composition data are available at the NIH Common Fund’s National Metabolomics Data Repository (NMDR) website, the Metabolomics Workbench, https://www.metabolomicsworkbench.org where it has been assigned Project ID (PR001116). The data can be accessed directly via it’s Project DOI: (10.21228/M8FM51).

### Lipid and bile acid measurements in tissues

Liver and cecal content lipids were extracted using Lipid Extraction Kit Chloroform Free (Abcam). Total and cholesterol ester (Millipore/Sigma-Aldrich), triglycerides (Abcam), free fatty acids (Abcam), and total bile acids (Total Bile Assay (NBT Method), GenWay Biotech Inc; San Diego, CA) were measured using the extracted liver lipids. Cecal content total bile acids were measured using the extracted cecal content lipids (Total Bile Assay (NBT Method) assay, GenWay Biotech Inc; San Diego, CA). Postprandial plasma obtained at termination of studies (see above for details) was used to measure total cholesterol (Pointe Scientific), triglycerides (Pointe Scientific), free fatty acids (Abcam), ALT (Pointe Scientific). All assays were performed according to the manufacturer’s instructions.

### Bone parameters

Tissues were fixed in 10% neutral-buffered formalin for 24 h and kept in Sorenson’s buffer (pH7.4) thereafter. Tibiae were placed in a 19-mm diameter specimen holder and scanned over the entire length of the tibiae using a microcomputed tomography (μCT) system (μCT100 Scanco Medical). Scan settings were as follows: voxel size 12 μm, 70 kVp, 114 μA, 0.5 mm AL filter, and integration time 500 ms. Density measurements were calibrated to the manufacturer’s hydroxyapatite phantom. Analysis was performed using the manufacturer’s evaluation software and a threshold of 180 for trabecular bone and 280 for cortical bone. Tibiae used for μCT scanning were decalcified in 14% EDTA for 3 weeks. Paraffin-embedded tissue sections were processed and stained with H&E.

Bone Marrow Adipose Tissue Quantification by Osmium Tetroxide Staining and μCT: Mouse tibiae were decalcified in 14% EDTA for 2-3 weeks, and then put into 1% osmium tetroxide solution (diluted by Sorenson’s buffer pH7.4) for 48 h. Osmium tetroxide-stained bones were scanned by the same program as described above. A threshold of 400 Gy was used for BMAT quantification. The volume of BMAT was normalized by the total volume (TV) of bone and shown as percentage (%).

### Muscle fiber area and ileal crypt depth/villi height analysis

Soleus muscle and ileal section were dissected and fixed in 10% neutral-buffered formalin overnight. Tissue was embedded in paraffin and sectioned onto slides and stained for H&E following standard protocol. Photos, and analysis of muscle fiber area (in 100–250 muscle fibers), and ileal crypt depth, and ileal villi height (in 25 villi and crypts) were acquired using Olympus IX73 fluorescence microscopy system (Olympus). Villus height was measured from the crypt-villus junction to the tip of the villus and crypt depth was measured from the base of the crypt to the crypt-villus junction. Images were analyzed using Olympus cellSens Standard imaging software (Olympus).

### Grip strength

Grip strength was measured with Columbus Instruments Grip Strength Meter (Colombus Instruments), which assesses neuromuscular function by sensing the peak amount of force an animal applies in grasping specially designed pull bar assemblies. Metering was performed with precision force gauges in such a manner as to retain the peak force applied on a digital display. The dual sensor model was employed by first allowing the animal to grasp the forelimb pull bar assembly. The animal was then drawn along a straight line leading away from the sensor. The animal released at some point and the maximum force attained is stored on the display. Each animal was tested five times and the average force reported in the data. Grip strength test was performed by the University of Michigan Physiology Phenotyping Core.

### Absorbed energy content

Fecal energy was assessed by the University of Michigan Animal Phenotyping core using Bomb Calorimeter, Parr 6200, and 1108P oxygen bomb^107^. Mice were single-housed in clean housing cages for one week. Food weight was determined for the same time period. All fecal samples are collected using forceps and weighed to determine “wet weight”. Prior to processing, fecal samples are dried overnight at 50 °C. Samples are then removed from the oven one at a time and weighed. Sample weights are recorded and fecal samples are then ground-up individually. Samples are ground with mortar and pestle and carefully scooped into a dry tube. All instruments were washed with sparkleen and 10% bleach and completely dried between samples. New weigh boats are used for each sample and weighed on the same scale as the pre-dried weights.

### Quantitative real-time PCR

RNA was extracted from tissue samples using RNeasy isolation kit (Qiagen). cDNA was synthesized by reverse transcription from mRNA using the iScript cDNA Synthesis Kit (Bio-Rad). Gene expression was performed by quantitative real-time RT-PCR using Taqman gene expression assay (Supplementary Table 1) and was performed using StepOnePlus detection system (Applied Biosystems) with a standard protocol. Relative abundance for each transcript was calculated by a standard curve of cycle thresholds and normalized to *RL32*. One Control VSG ileum sample was excluded due to the high cycle threshold of *RL32* (Ct over 30 after two independent measurements).

### Intestinal biometry

Following euthanasia, the entire gastrointestinal tract from the stomach to the rectum was removed, cleaned of mesenteric fat and gut weight and length determined. Small and large intestine/colon length was measured on a horizontal ruler after flushing with PBS. The entire small and large intestine/colon were then blotted to remove PBS before being weighed.

### Analysis of 16S rRNA Gene Sequences

*16S rRNA Sequencing:* Cecal contents were added to individual Bead plate provided by the Microbiome Core at the University of Michigan. DNA was isolated using Qiagen MagAttract PowerMicrobiome kit DNA/RNA kit (Qiagen, catalog no. 27500-4-EP) on the EpMotion 5075 (Eppendorf) liquid handler. Extracted DNA was then used to generate 16S rRNA libraries for community analysis. The DNA libraries were prepared by the Microbiome Core as described previously^108^. Briefly, DNA was PCR amplified using a set of barcoded dual-index primers specific to the V4 region of the 16S rRNA gene^109^. PCR reactions are composed of 5 µL of 4 µM equimolar primer set, 0.15 µL of AccuPrime Taq DNA High Fidelity Polymerase, 2 µL of 10x AccuPrime PCR Buffer II (Thermo Fisher Scientific, catalog no.12346094), 11.85 µL of PCR-grade water, and 1 µL of DNA template. The PCR conditions used consisted of 2 min at 95 °C, followed by 30 cycles of 95 °C for 20 s, 55 °C for 15 s, and 72 °C for 5 min, followed by 72 °C for 10 min. Each PCR reaction is normalized using the SequalPrep Normalization Plate Kit (Thermo Fisher Scientific, catalog no. A1051001). The normalized reactions are pooled and quantified using the Kapa Biosystems Library qPCR MasterMix (ROX Low) Quantification kit for Illumina platforms (catalog no. KK4873). The Agilent Bioanalyzer is used to confirm the size of the amplicon library (~399 bp) using a high-sensitive DNA analysis kit (catalog no. 5067-4626). Pooled amplicon library is then sequenced on the Illumina MiSeq platform using the 500 cycle MiSeq V2 Reagent kit (catalog no. MS-102-2003) according to the manufacturer’s instructions with modifications of the primer set with custom read 1/read 2 and index primers added to the reagent cartridge. The “Preparing Libraries for Sequencing on the MiSeq” (part 15039740, Rev. D) protocol was used to prepare libraries with a final load concentration of 5.5 pM, spiked with 15% PhiX to create diversity within the run. FASTQ files are generated when the 2 × 250 bp sequencing completes. *Analysis:* Following sequencing, microbiome bioinformatics were run using QIIME 2 2020.2^110^. Briefly, non-singleton amplicon sequence variants (ASVs, 100% operational taxonomic units (OTUs)) were generated from raw sequences after trimming with the cutadapt plugin denoising with the dada2 plugin. One Control VSG and one FGF15^INT-KO^ Sham samples were excluded because of low OTUs. Taxonomy was then assigned to ASVs using the classify-sklearn alignment algorithm (Bokulich et al.^111^) against the Greengenes database (Release 13.8) of 99% OTUs reference sequences (McDonald et al.^112^). Alpha diversity metrics including Chao1 and Shannon, which estimate with-in sample richness and diversity respectively, were calculated using the diversity plugin. Chao1 index represents the number of ASVs present in one single sample, while Shannon index accounts for both abundance and evenness of ASVs present. Beta diversity metrics including weighted and unweighted UniFrac distance matrix^113^, which estimate between-sample dissimilarity, were scaled and visualized through principal coordinates analysis (PCoA), and further used to determine the significance of the clustering between groups via permutational multivariate analysis of variance (PERMANOVA). Linear discriminant analysis (LDA) effect size (LEfSe) with default parameters^114^ and Random Forest Classifier (QIIME 2 2020.2) with 10-fold cross-validations^115^ were computed to identify significantly different microbes in abundance between groups at different taxonomic levels. The 16S rRNA Sequencing composition data generated in this study have been deposited in the Sequence Read Archive (SRA) where it has been assigned BioProject ID PRJNA734599. The data can be accessed directly via it’s Project URL: http://www.ncbi.nlm.nih.gov/bioproject/734599.

### Statistics and reproducibility

The researchers were blinded during studies and mouse order (per genotype and surgery) were randomized. Microcomputed tomography (μCT) analysis for bone parameters and bone marrow adiposity were completed by two independent experienced lab researchers in a blinded manner. Bone histology images were taken from each individual mouse and all images were quantified. Representative images of the mean number based on the quantification were shown in Fig. 2. Liver histology images were taken from each individual mouse and representative images were shown in Fig. 3. All metabolic studies were performed at least twice with the same results and data were shown over multiple time points (body weight, body composition, food intake, glucose tolerance). All studies were performed in a sufficient number of biological replicates per genotype and surgery.

### Statistical analysis

The statistical analysis for comparisons between two groups was performed by unpaired (two-tailed) Student’s *t* test. Two-way ANOVA with post hoc Tukey’s multiple comparisons post hoc test was used for comparisons among four groups. *P* values <0.05 were considered significant. Statistical analysis was performed using GraphPad Prism 8.2.0. Microbiome analysis is described above.

### Reporting summary

Further information on research design is available in the Nature Research Reporting Summary linked to this article.

## Supplementary information

Supplementary Information

Reporting Summary

## Data Availability

Plasma bile acid composition data generated in this study have been deposited in the NIH Common Fund’s National Metabolomics Data Repository (NMDR) website, the Metabolomics Workbench, https://www.metabolomicsworkbench.org where it has been assigned Project ID (PR001116). The data can be accessed directly via it’s Project DOI: (10.21228/M8FM51). The 16S rRNA Sequencing composition data generated in this study have been deposited in the Sequence Read Archive (SRA) where it has been assigned BioProject ID PRJNA734599. The data can be accessed directly via it’s Project URL: http://www.ncbi.nlm.nih.gov/bioproject/734599. The authors declare that the data supporting the findings of this study are available within the paper and its supplementary information files. Source data are provided with this paper.

## Source data

Source Data

## References

[1] Afshin A, Reitsma MB, Murray CJL (2017). Health effects of overweight and obesity in 195 countries. N. Engl. J. Med..

[2] Saltiel AR (2016). New therapeutic approaches for the treatment of obesity. Sci. Transl. Med..

[3] Adams TD (2017). Weight and metabolic outcomes 12 years after gastric bypass. N. Engl. J. Med..

[4] Schauer PR (2017). Bariatric surgery versus intensive medical therapy for diabetes - 5-year outcomes. N. Engl. J. Med..

[5] Mingrone G (2021). Metabolic surgery versus conventional medical therapy in patients with type 2 diabetes: 10-year follow-up of an open-label, single-centre, randomised controlled trial. Lancet.

[6] Myronovych A (2014). Vertical sleeve gastrectomy reduces hepatic steatosis while increasing serum bile acids in a weight-loss-independent manner. Obesity.

[7] Kohli R (2010). Intestinal adaptation after ileal interposition surgery increases bile acid recycling and protects against obesity-related comorbidities. Am. J. Physiol. Gastrointest. Liver Physiol..

[8] Patti ME (2009). Serum bile acids are higher in humans with prior gastric bypass: potential contribution to improved glucose and lipid metabolism. Obesity.

[9] Pournaras DJ (2012). The role of bile after Roux-en-Y gastric bypass in promoting weight loss and improving glycaemic control. Endocrinology.

[10] Bozadjieva N, Heppner KM, Seeley RJ (2018). Targeting FXR and FGF19 to treat metabolic diseases-lessons learned from bariatric surgery. Diabetes.

[11] Pournaras DJ, le Roux CW (2013). Are bile acids the new gut hormones? Lessons from weight loss surgery models. Endocrinology.

[12] Ryan KK (2014). FXR is a molecular target for the effects of vertical sleeve gastrectomy. Nature.

[13] Albaugh VL (2019). Role of bile acids and GLP-1 in mediating the metabolic improvements of bariatric surgery. Gastroenterology.

[14] Inagaki T (2005). Fibroblast growth factor 15 functions as an enterohepatic signal to regulate bile acid homeostasis. Cell Metab..

[15] Fon Tacer K (2010). Research resource: comprehensive expression atlas of the fibroblast growth factor system in adult mouse. Mol. Endocrinol..

[16] Somm E, Jornayvaz FR (2018). Fibroblast growth factor 15/19: from basic functions to therapeutic perspectives. Endocr. Rev..

[17] Morton GJ, Kaiyala KJ, Foster-Schubert KE, Cummings DE, Schwartz MW (2014). Carbohydrate feeding dissociates the postprandial FGF19 response from circulating bile acid levels in humans. J. Clin. Endocrinol. Metab..

[18] Kir S (2011). FGF19 as a postprandial, insulin-independent activator of hepatic protein and glycogen synthesis. Science.

[19] Potthoff MJ (2011). FGF15/19 regulates hepatic glucose metabolism by inhibiting the CREB-PGC-1alpha pathway. Cell Metab..

[20] Lan T (2017). FGF19, FGF21, and an FGFR1/beta-Klotho-activating antibody act on the nervous system to regulate body weight and glycemia. Cell Metab..

[21] Harrison SA (2018). NGM282 for treatment of non-alcoholic steatohepatitis: a multicentre, randomised, double-blind, placebo-controlled, phase 2 trial. Lancet.

[22] DePaoli AM (2019). FGF19 analog as a surgical factor mimetic that contributes to metabolic effects beyond glucose homeostasis. Diabetes.

[23] Harrison SA (2020). NGM282 improves liver fibrosis and histology in 12 weeks in patients with nonalcoholic steatohepatitis. Hepatology.

[24] Fu L (2004). Fibroblast growth factor 19 increases metabolic rate and reverses dietary and leptin-deficient diabetes. Endocrinology.

[25] Tomlinson E (2002). Transgenic mice expressing human fibroblast growth factor-19 display increased metabolic rate and decreased adiposity. Endocrinology.

[26] Morton GJ (2013). FGF19 action in the brain induces insulin-independent glucose lowering. J. Clin. Invest..

[27] Ryan KK (2013). Fibroblast growth factor-19 action in the brain reduces food intake and body weight and improves glucose tolerance in male rats. Endocrinology.

[28] Miyata M, Sakaida Y, Matsuzawa H, Yoshinari K, Yamazoe Y (2011). Fibroblast growth factor 19 treatment ameliorates disruption of hepatic lipid metabolism in farnesoid X receptor (Fxr)-null mice. Biol. Pharm. Bull..

[29] Mraz M (2011). Serum concentrations of fibroblast growth factor 19 in patients with obesity and type 2 diabetes mellitus: the influence of acute hyperinsulinemia, very-low calorie diet and PPAR-alpha agonist treatment. Physiol. Res..

[30] Gallego-Escuredo JM (2015). Opposite alterations in FGF21 and FGF19 levels and disturbed expression of the receptor machinery for endocrine FGFs in obese patients. Int. J. Obes..

[31] Renner O (2014). Upregulation of hepatic bile acid synthesis via fibroblast growth factor 19 is defective in gallstone disease but functional in overweight individuals. U. Eur. Gastroenterol. J..

[32] Gomez-Ambrosi J (2017). FGF19 and FGF21 serum concentrations in human obesity and type 2 diabetes behave differently after diet- or surgically-induced weight loss. Clin. Nutr..

[33] Haluzikova D (2013). Laparoscopic sleeve gastrectomy differentially affects serum concentrations of FGF-19 and FGF-21 in morbidly obese subjects. Obesity.

[34] Sonne DP (2016). Postprandial plasma concentrations of individual bile acids and FGF-19 in patients with type 2 diabetes. J. Clin. Endocrinol. Metab..

[35] Barutcuoglu B (2011). Fibroblast growth factor-19 levels in type 2 diabetic patients with metabolic syndrome. Ann. Clin. Lab Sci..

[36] Eren F (2012). Preliminary evidence of a reduced serum level of fibroblast growth factor 19 in patients with biopsy-proven nonalcoholic fatty liver disease. Clin. Biochem..

[37] Jiao N (2018). Suppressed hepatic bile acid signalling despite elevated production of primary and secondary bile acids in NAFLD. Gut.

[38] Mulla CM (2019). Plasma FGF-19 levels are increased in patients with post-bariatric hypoglycemia. Obes. Surg..

[39] Sachdev S (2016). FGF 19 and bile acids increase following Roux-en-Y gastric bypass but not after medical management in patients with type 2 diabetes. Obes. Surg..

[40] Belgaumkar AP (2016). Changes in bile acid profile after laparoscopic sleeve gastrectomy are associated with improvements in metabolic profile and fatty liver disease. Obes. Surg..

[41] Angelin B, Larsson TE, Rudling M (2012). Circulating fibroblast growth factors as metabolic regulators–a critical appraisal. Cell Metab..

[42] Montagnani M (2011). A new model for portal protein profile analysis in course of ileal intraluminal bile acid infusion using an in situ perfused rat intestine. Med Chem..

[43] Myronovych, A. et al. Assessment of the role of FGF15 in mediating the metabolic outcomes of murine Vertical Sleeve Gastrectomy (VSG). *Am. J. Physiol. Gastrointest. Liver. Physiol*. 10.1152/ajpgi.00175.2020 (2020). 10.1152/ajpgi.00175.2020 PMC7792670 32967428

[44] Gimeno L, Brulet P, Martinez S (2003). Study of Fgf15 gene expression in developing mouse brain. Gene Expr. Patterns.

[45] Gimeno L, Hashemi R, Brulet P, Martinez S (2002). Analysis of Fgf15 expression pattern in the mouse neural tube. Brain Res. Bull..

[46] McWhirter JR, Goulding M, Weiner JA, Chun J, Murre C (1997). A novel fibroblast growth factor gene expressed in the developing nervous system is a downstream target of the chimeric homeodomain oncoprotein E2A-Pbx1. Development.

[47] Picard A (2016). A genetic screen identifies hypothalamic Fgf15 as a regulator of glucagon secretion. Cell Rep..

[48] Patel A (2018). GLP-2 receptor signaling controls circulating bile acid levels but not glucose homeostasis in Gcgr(-/-) mice and is dispensable for the metabolic benefits ensuing after vertical sleeve gastrectomy. Mol. Metab..

[49] Benoit B (2017). Fibroblast growth factor 19 regulates skeletal muscle mass and ameliorates muscle wasting in mice. Nat. Med.

[50] Oost LJ, Kustermann M, Armani A, Blaauw B, Romanello V (2019). Fibroblast growth factor 21 controls mitophagy and muscle mass. J. Cachexia Sarcopenia Muscle.

[51] Mao J (2013). Overnutrition stimulates intestinal epithelium proliferation through beta-catenin signaling in obese mice. Diabetes.

[52] Li Z (2019). G-CSF partially mediates effects of sleeve gastrectomy on the bone marrow niche. J. Clin. Invest..

[53] Scheller EL (2015). Region-specific variation in the properties of skeletal adipocytes reveals regulated and constitutive marrow adipose tissues. Nat. Commun..

[54] Daryadel A (2019). Elevated FGF23 and disordered renal mineral handling with reduced bone mineralization in chronically erythropoietin over-expressing transgenic mice. Sci. Rep..

[55] Hiram-Bab S (2015). Erythropoietin directly stimulates osteoclast precursors and induces bone loss. FASEB J..

[56] Clinkenbeard EL (2017). Erythropoietin stimulates murine and human fibroblast growth factor-23, revealing novel roles for bone and bone marrow. Haematologica.

[57] Liou AP (2013). Conserved shifts in the gut microbiota due to gastric bypass reduce host weight and adiposity. Sci. Transl. Med..

[58] Sanmiguel CP (2017). Surgically induced changes in gut microbiome and hedonic eating as related to weight loss: preliminary findings in obese women undergoing bariatric surgery. Psychosom. Med..

[59] Basso N (2016). Insulin resistance, microbiota, and fat distribution changes by a new model of vertical sleeve gastrectomy in obese rats. Diabetes.

[60] Wells JM, Mercenier A (2008). Mucosal delivery of therapeutic and prophylactic molecules using lactic acid bacteria. Nat. Rev. Microbiol..

[61] Crovesy L, Ostrowski M, Ferreira D, Rosado EL, Soares-Mota M (2017). Effect of Lactobacillus on body weight and body fat in overweight subjects: a systematic review of randomized controlled clinical trials. Int J. Obes..

[62] Kim I (2007). Differential regulation of bile acid homeostasis by the farnesoid X receptor in liver and intestine. J. Lipid Res..

[63] Jiang C (2015). Intestine-selective farnesoid X receptor inhibition improves obesity-related metabolic dysfunction. Nat. Commun..

[64] Li F (2013). Microbiome remodelling leads to inhibition of intestinal farnesoid X receptor signalling and decreased obesity. Nat. Commun..

[65] Prawitt J (2011). Farnesoid X receptor deficiency improves glucose homeostasis in mouse models of obesity. Diabetes.

[66] Baker DM, Wang SL, Bell DJ, Drevon CA, Davis RA (2000). One or more labile proteins regulate the stability of chimeric mRNAs containing the 3’-untranslated region of cholesterol-7alpha -hydroxylase mRNA. J. Biol. Chem..

[67] Agellon LB, Cheema SK (1997). The 3’-untranslated region of the mouse cholesterol 7alpha-hydroxylase mRNA contains elements responsive to post-transcriptional regulation by bile acids. Biochem J..

[68] Schadt HS (2018). Bile acid sequestration by cholestyramine mitigates FGFR4 inhibition-induced ALT elevation. Toxicol. Sci..

[69] Perez MJ, Briz O (2009). Bile-acid-induced cell injury and protection. World J. Gastroenterol..

[70] Pezeshki A, Zapata RC, Singh A, Yee NJ, Chelikani PK (2016). Low protein diets produce divergent effects on energy balance. Sci. Rep..

[71] Inagaki T (2007). Endocrine regulation of the fasting response by PPARalpha-mediated induction of fibroblast growth factor 21. Cell Metab..

[72] Badman MK (2007). Hepatic fibroblast growth factor 21 is regulated by PPARalpha and is a key mediator of hepatic lipid metabolism in ketotic states. Cell Metab..

[73] Khan FH (2016). Fibroblast growth factor 21 correlates with weight loss after vertical sleeve gastrectomy in adolescents. Obesity.

[74] Dushay J (2010). Increased fibroblast growth factor 21 in obesity and nonalcoholic fatty liver disease. Gastroenterology.

[75] Zhang X (2008). Serum FGF21 levels are increased in obesity and are independently associated with the metabolic syndrome in humans. Diabetes.

[76] Li H (2010). Fibroblast growth factor 21 levels are increased in nonalcoholic fatty liver disease patients and are correlated with hepatic triglyceride. J. Hepatol..

[77] Kim SH (2015). Fibroblast growth factor 21 participates in adaptation to endoplasmic reticulum stress and attenuates obesity-induced hepatic metabolic stress. Diabetologia.

[78] Tezze C, Romanello V, Sandri M (2019). FGF21 as modulator of metabolism in health and disease. Front. Physiol..

[79] Wei W (2012). Fibroblast growth factor 21 promotes bone loss by potentiating the effects of peroxisome proliferator-activated receptor gamma. Proc. Natl Acad. Sci. USA.

[80] Fazeli PK (2015). FGF21 and the late adaptive response to starvation in humans. J. Clin. Invest..

[81] Bornstein S (2014). FGF-21 and skeletal remodeling during and after lactation in C57BL/6J mice. Endocrinology.

[82] Lee JM (2018). Diet1, bile acid diarrhea, and FGF15/19: mouse model and human genetic variants. J. Lipid Res..

[83] Farr S (2020). Bile acid treatment and FXR agonism lower postprandial lipemia in mice. Am. J. Physiol. Gastrointest. Liver Physiol..

[84] Jorgensen NB (2015). Improvements in glucose metabolism early after gastric bypass surgery are not explained by increases in total bile acids and fibroblast growth factor 19 concentrations. J. Clin. Endocrinol. Metab..

[85] Thomas C (2009). TGR5-mediated bile acid sensing controls glucose homeostasis. Cell Metab..

[86] Potthoff MJ (2013). Colesevelam suppresses hepatic glycogenolysis by TGR5-mediated induction of GLP-1 action in DIO mice. Am. J. Physiol. Gastrointest. Liver Physiol..

[87] Katsuma S, Hirasawa A, Tsujimoto G (2005). Bile acids promote glucagon-like peptide-1 secretion through TGR5 in a murine enteroendocrine cell line STC-1. Biochem. Biophys. Res. Commun..

[88] Brighton CA (2015). Bile acids trigger GLP-1 release predominantly by accessing basolaterally located G protein-coupled bile acid receptors. Endocrinology.

[89] Bozadjieva Kramer N (2020). The role of elevated branched-chain amino acids in the effects of vertical sleeve gastrectomy to reduce weight and improve glucose regulation. Cell Rep..

[90] Gregory NS (2017). The effects of bariatric surgery on bone metabolism. Endocrinol. Metab. Clin. North Am..

[91] Stein EM, Silverberg SJ (2014). Bone loss after bariatric surgery: causes, consequences, and management. Lancet Diabetes Endocrinol..

[92] Choksi P (2018). Weight loss and bone mineral density in obese adults: a longitudinal analysis of the influence of very low energy diets. Clin. Diabetes Endocrinol..

[93] Kenngott HG (2019). Weight loss and changes in adipose tissue and skeletal muscle volume after laparoscopic sleeve gastrectomy and Roux-en-Y gastric bypass: a prospective study with 12-month follow-up. Obes. Surg..

[94] Kim, J., Brethauer, S. & Committee, A. C. I., American Society for, M. & Bariatric Surgery Clinical Issues Committee, P. S. Metabolic bone changes after bariatric surgery. *Surg. Obes. Relat. Dis*. **11**, 406–411 (2015). 10.1016/j.soard.2014.03.010 25487633

[95] Kolomansky, A. et al. Erythropoietin mediated bone loss in mice is dose-dependent and mostly irreversible. *Int. J. Mol. Sci.*10.3390/ijms21113817 (2020). 10.3390/ijms21113817 PMC7312352 32471308

[96] Arble DM (2018). Metabolic comparison of one-anastomosis gastric bypass, single-anastomosis duodenal-switch, Roux-en-Y gastric bypass, and vertical sleeve gastrectomy in rat. Surg. Obes. Relat. Dis..

[97] Ashby K (2018). Review article: therapeutic bile acids and the risks for hepatotoxicity. Aliment Pharm. Ther..

[98] Attili AF, Angelico M, Cantafora A, Alvaro D, Capocaccia L (1986). Bile acid-induced liver toxicity: relation to the hydrophobic-hydrophilic balance of bile acids. Med. Hypotheses.

[99] Qiu Y (2021). Depletion of gut microbiota induces skeletal muscle atrophy by FXR-FGF15/19 signalling. Ann. Med..

[100] Desai MS (2017). Bile acid excess induces cardiomyopathy and metabolic dysfunctions in the heart. Hepatology.

[101] Vasavan T (2018). Heart and bile acids - Clinical consequences of altered bile acid metabolism. Biochim Biophys. Acta Mol. Basis Dis..

[102] Faul C (2011). FGF23 induces left ventricular hypertrophy. J. Clin. Invest..

[103] Manolis AS (2005). Erythropoietin in heart failure and other cardiovascular diseases: hematopoietic and pleiotropic effects. Curr. Drug Targets Cardiovasc. Haematol. Disord..

[104] Kim, K. S. et al. Glycemic effect of pancreatic preproglucagon in mouse sleeve gastrectomy. *JCI Insight*10.1172/jci.insight.129452 (2019). 10.1172/jci.insight.129452 PMC6824314 31619587

[105] Evers, S. S. et al. The unconventional role for gastric volume in the response to bariatric surgery for both weight loss and glucose lowering. *Ann. Surg*. 10.1097/SLA.0000000000003240 (2019). 10.1097/SLA.0000000000003240 30817350

[106] Griffiths WJ, Sjovall J (2010). Bile acids: analysis in biological fluids and tissues. J. Lipid Res..

[107] Asai M (2013). Loss of function of the melanocortin 2 receptor accessory protein 2 is associated with mammalian obesity. Science.

[108] Seekatz AM (2015). Fecal microbiota transplantation eliminates *Clostridium difficile* in a murine model of relapsing disease. Infect. Immun..

[109] Kozich JJ, Westcott SL, Baxter NT, Highlander SK, Schloss PD (2013). Development of a dual-index sequencing strategy and curation pipeline for analyzing amplicon sequence data on the MiSeq Illumina sequencing platform. Appl. Environ. Microbiol..

[110] Bolyen E (2019). Reproducible, interactive, scalable and extensible microbiome data science using QIIME 2. Nat. Biotechnol..

[111] Bokulich NA (2018). Optimizing taxonomic classification of marker-gene amplicon sequences with QIIME 2’s q2-feature-classifier plugin. Microbiome.

[112] McDonald D (2012). An improved Greengenes taxonomy with explicit ranks for ecological and evolutionary analyses of bacteria and archaea. ISME J.

[113] Lozupone CA, Hamady M, Kelley ST, Knight R (2007). Quantitative and qualitative beta diversity measures lead to different insights into factors that structure microbial communities. Appl. Environ. Microbiol..

[114] Segata N (2011). Metagenomic biomarker discovery and explanation. Genome Biol..

[115] Breiman L (2001). Random forests. Machone Learn..

