# Supplementary material for: Intestinal-derived FGF15 protects against deleterious effects of vertical sleeve gastrectomy in mice
Source: Nat Commun. 2021 Aug 6;12:4768. doi: 10.1038/s41467-021-24914-y (PMC8346483; doi:10.1038/s41467-021-24914-y)
Supplement: Supplementary file 1 — Supplementary Information [file 41467_2021_24914_MOESM1_ESM.pdf]

Supplementary Figures and Table for Bozadjieva-Kramer et. al.

Supplementary Figure 1

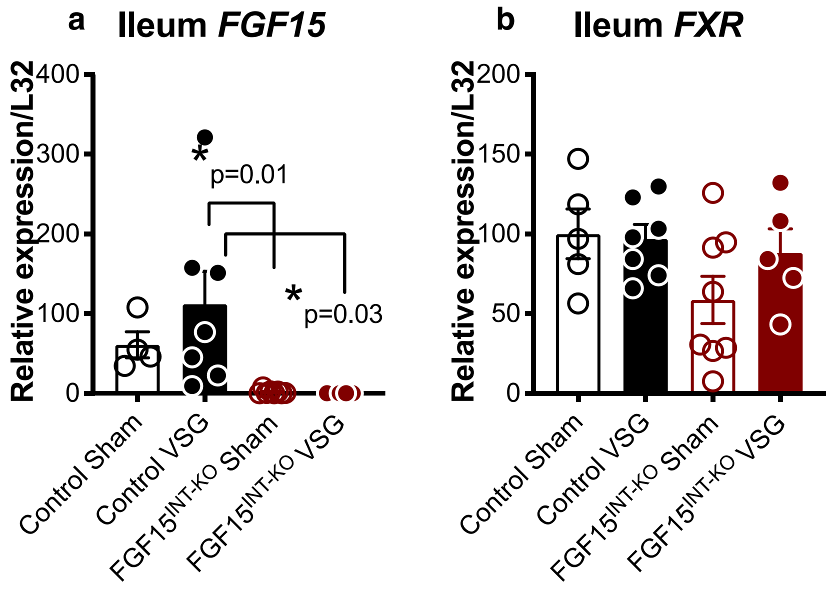

Supplementary Figure 1. The RNA expression of ileal FGF15 is ablated in FGF15^{INT-KO} mice. a. RNA expression of *FGF15* in ileum. Animal number Control Sham (n=4), Control VSG (n=7), FGF15^{INT-KO} Sham (n=8), FGF15^{INT-KO} VSG (n=5). **b.** RNA expression of *FXR* in ileum. Animal number Control Sham (n=5), Control VSG (n=7), FGF15^{INT-KO} Sham (n=8), FGF15^{INT-KO} VSG (n=5). Data are shown as means ± S.E.M. *$p<0.05$ (2-Way ANOVA with Tukey's post-hoc test).

Supplementary Figure 2

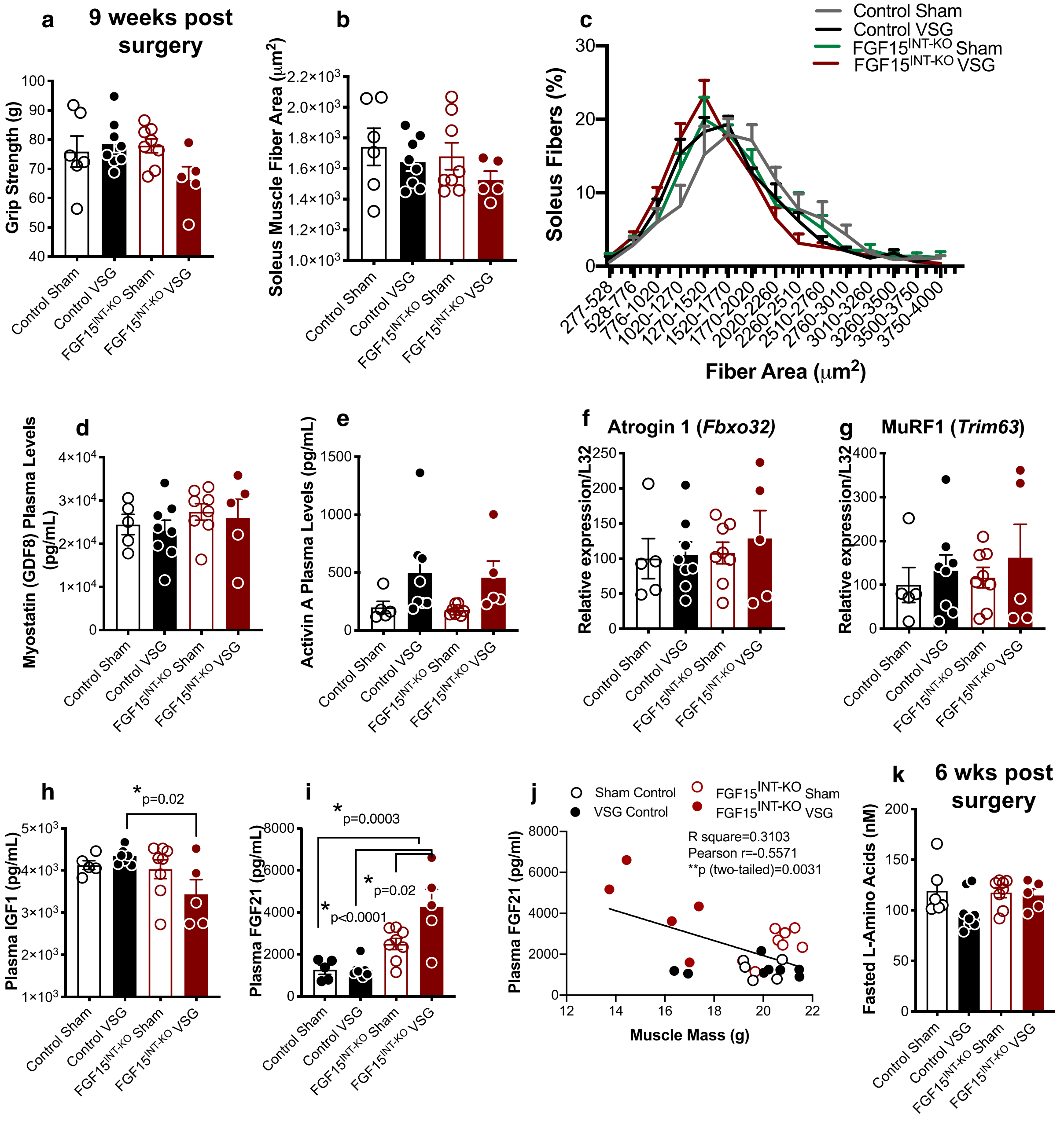

Supplementary Figure 2. Loss of muscle mass is accompanied by decreased strength and skeletal muscle fiber size distribution in FGF15^{INT-KO} VSG mice. a. Grip strength measured 9 weeks post-surgery. **b.** Soleus muscle average fiber area. **c.** Soleus fiber size distribution. **d.** Myostatin (GDF8) and **e.** Activin A plasma levels (postprandial, 12 weeks after surgery). RNA expression of **f.** Atrogin-1 (encoded by *Fbxo32*) and **g.** MuRF1 (encoded by *Trim63*) in soleus muscle. **h.** IGF-1 and **i.** FGF21 plasma levels (postprandial, 12 weeks after surgery). **j.** Correlation analysis of plasma FGF21 levels and muscle mass (12 weeks after surgery). **k.** Fasting L-Amino Acid levels in plasma, 6 weeks post-surgery. Animal number for a-c and k: Control Sham (n=6), Control VSG (n=8), FGF15^{INT-KO} Sham (n=8), FGF15^{INT-KO} VSG (n=5). Animal number for d-j: Control Sham (n=5), Control VSG (n=8), FGF15^{INT-KO} Sham (n=8), FGF15^{INT-KO} VSG (n=5). Data are shown as means ± S.E.M. *p<0.05 (2-Way ANOVA with Tukey's post-hoc test).

Supplementary Figure 3

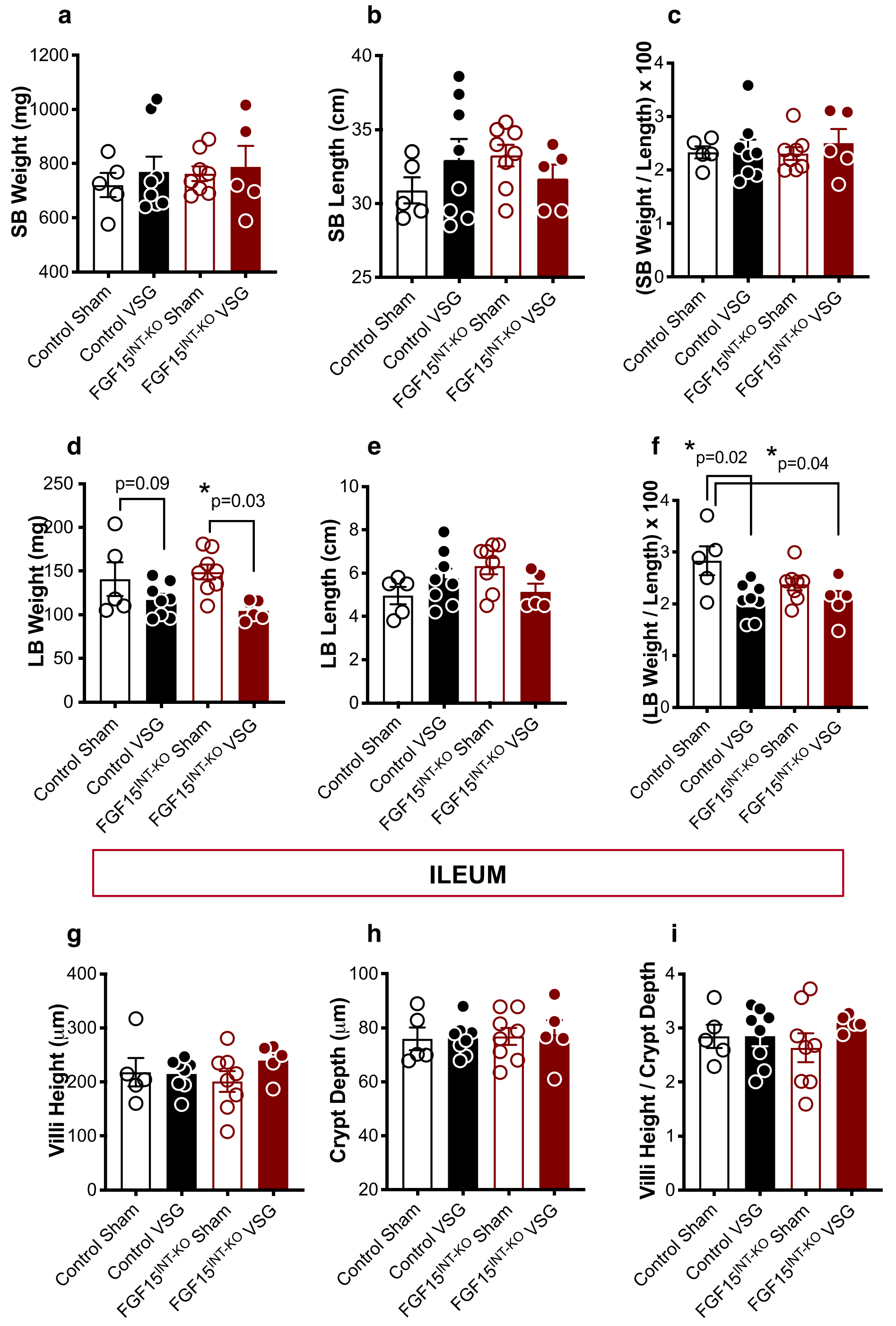

Supplementary Figure 3. Intestinal biometry in mice lacking intestinal-derived FGF15. SB: small bowel, LB: large bowel. **a.** SB weight. **b.** SB length. **c.** SB Weight/Length. **d.** LB weight. **e.** LB length. **f.** LB Weight/Length. **g.** Ileum villi height. **h.** Ileum crypt depth. **i.** Ileum villi height/crypt depth. Animal number Control Sham (n=5), Control VSG (n=8), $FGF15^{INT\text{-}KO}$ Sham (n=8), $FGF15^{INT\text{-}KO}$ VSG (n=5). Data are shown as means ± S.E.M. *p<0.05 (2-Way ANOVA with Tukey's post-hoc test).

Supplementary Figure 4

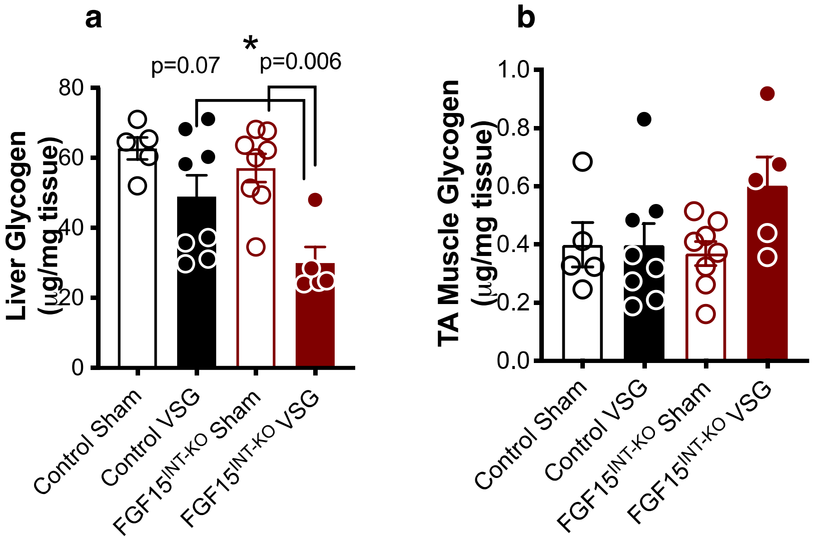

Supplementary Figure 4. Loss of intestinal FGF15 results in aberrant glycogen metabolism following VSG. a. Liver glycogen content and **b.** Muscle glycogen content (TA; tibialis anterior). Animal number Control Sham (n=5), Control VSG (n=8), FGF15^{INT-KO} Sham (n=8), FGF15^{INT-KO} VSG (n=5). Data are shown as means ± S.E.M. *p<0.05 (2-Way ANOVA with Tukey's post-hoc test).

Supplementary Figure 5

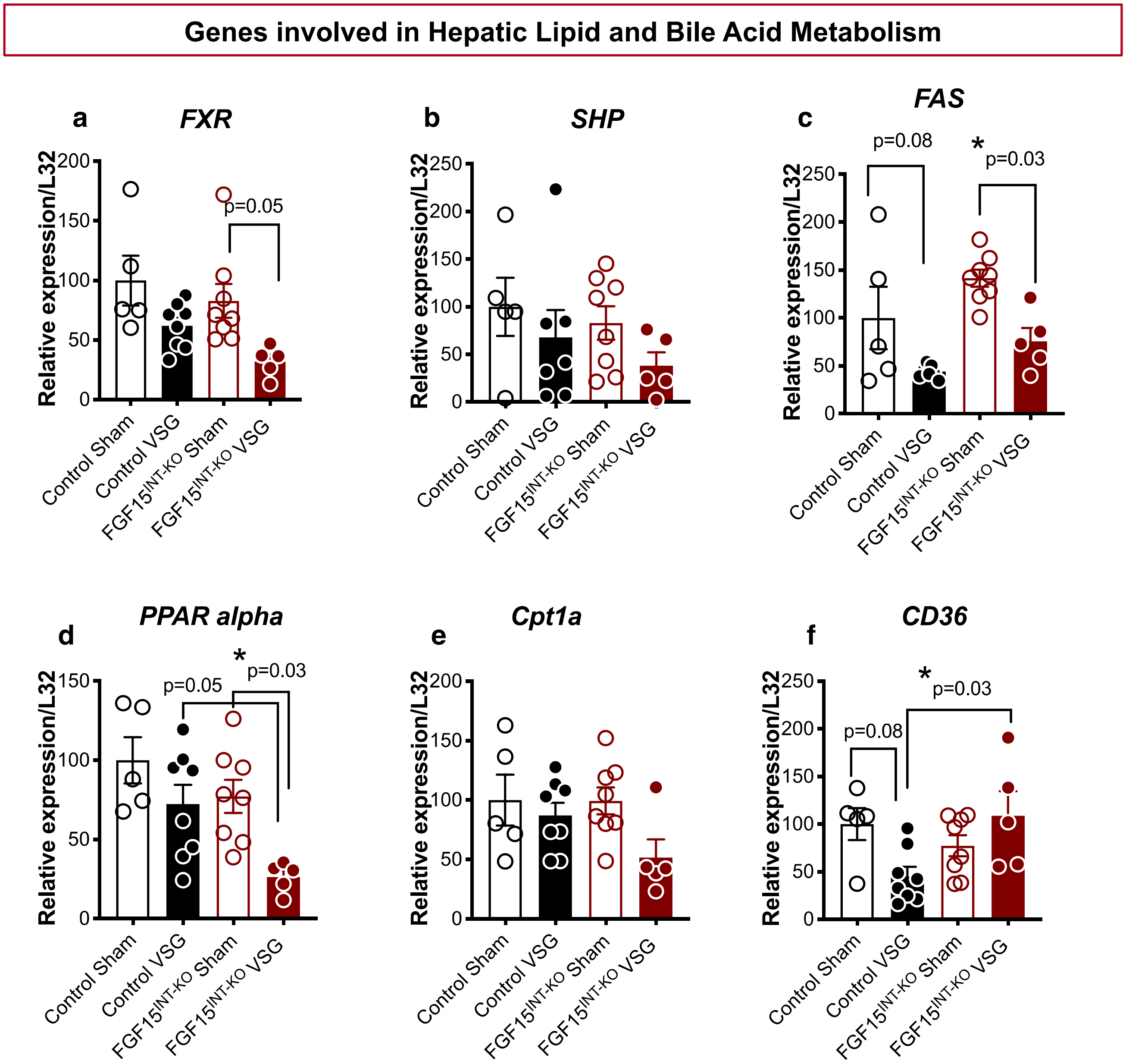

Supplementary Figure 5. Hepatic fatty acid and lipid metabolism. Hepatic RNA expression of **a.** Farnesoid X receptor (*FXR*), **b.** Small heterodimer partner (*SHP*), **c.** Fatty acid synthase (*FAS*) **d.** Peroxisome proliferator-activated receptor alpha (*PPAR alpha*), **e.** Carnitine palmitoyltransferase 1A (*Cpt1a*) and **f.** Cluster of differentiation 36 (*CD36*). Animal number for a, d-f: Control Sham (n=5), Control VSG (n=8), FGF15^{INT-KO} Sham (n=8), FGF15^{INT-KO} VSG (n=5). Animal number for b and c: Control Sham (n=5), Control VSG (n=7), FGF15^{INT-KO} Sham (n=8), FGF15^{INT-KO} VSG (n=5). Data are shown as means ± S.E.M. *p<0.05 (2-Way ANOVA with Tukey's post-hoc test).

Supplementary Figure 6

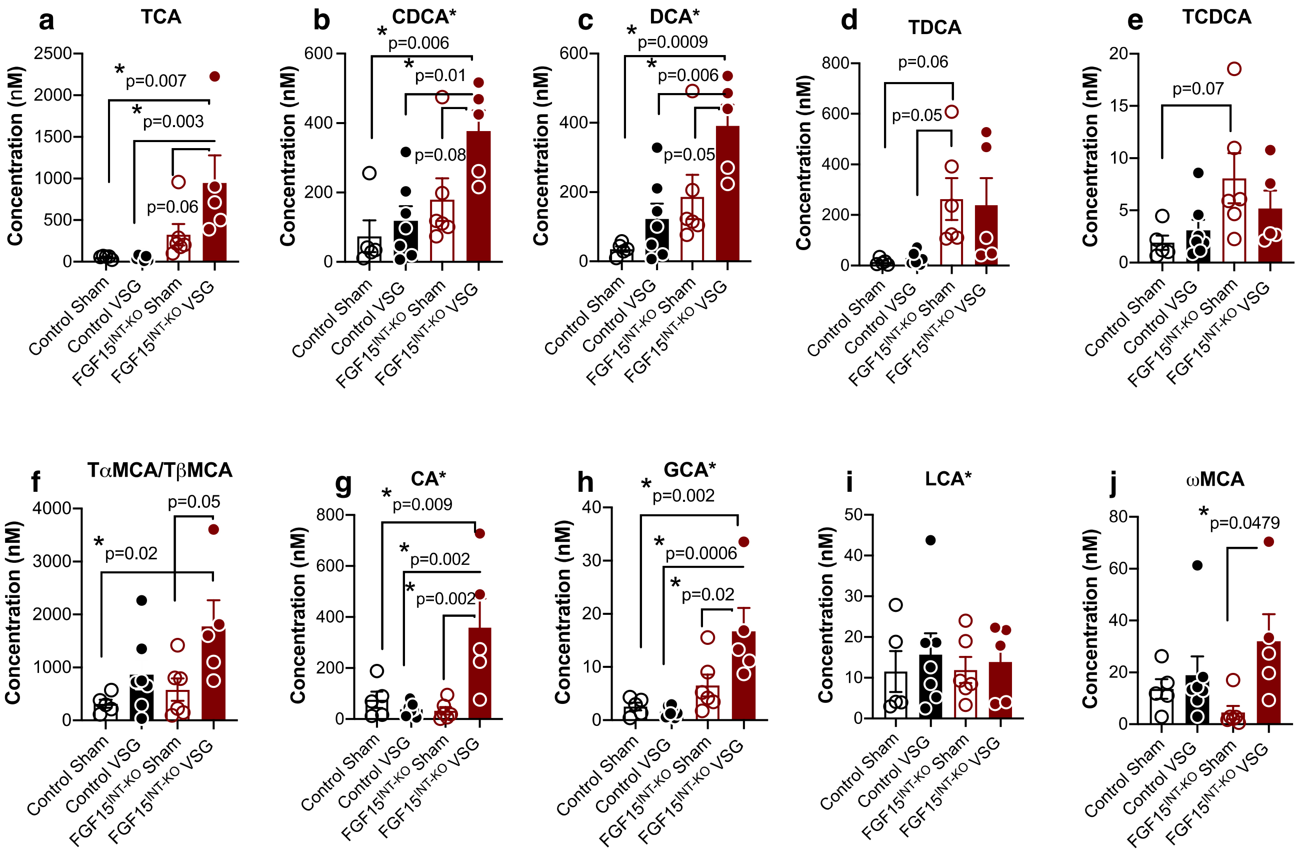

Supplementary Figure 6. Plasma bile acid composition. a. Taurocholic acid (TCA) **b.** Chenodeoxycholic acid (CDCA) **c.** Deoxycholic acid (DCA) **d.** Taurodeoxycholic acid (TDCA) **e.** Taurochenodeoxycholic acid (TCDCA) **f.** TαMCA (tauro-α muricholate)/TβMCA (tauro-β-muricholate) **g.** Cholate (CA) **h.** Glycocholate acid (GCA) **i.** Lithocholic acid (LCA) **j.** Muricholic acid (ωMCA). Animal number Control Sham (n=5), Control VSG (n=7), FGF15^{INT-KO} Sham (n=6), FGF15^{INT-KO} VSG (n=5). Data are shown as means ± S.E.M. *p<0.05 (2-Way ANOVA with Tukey's post-hoc test). *next to bile acid name denotes hydrophobic bile acid

Supplementary Figure 7

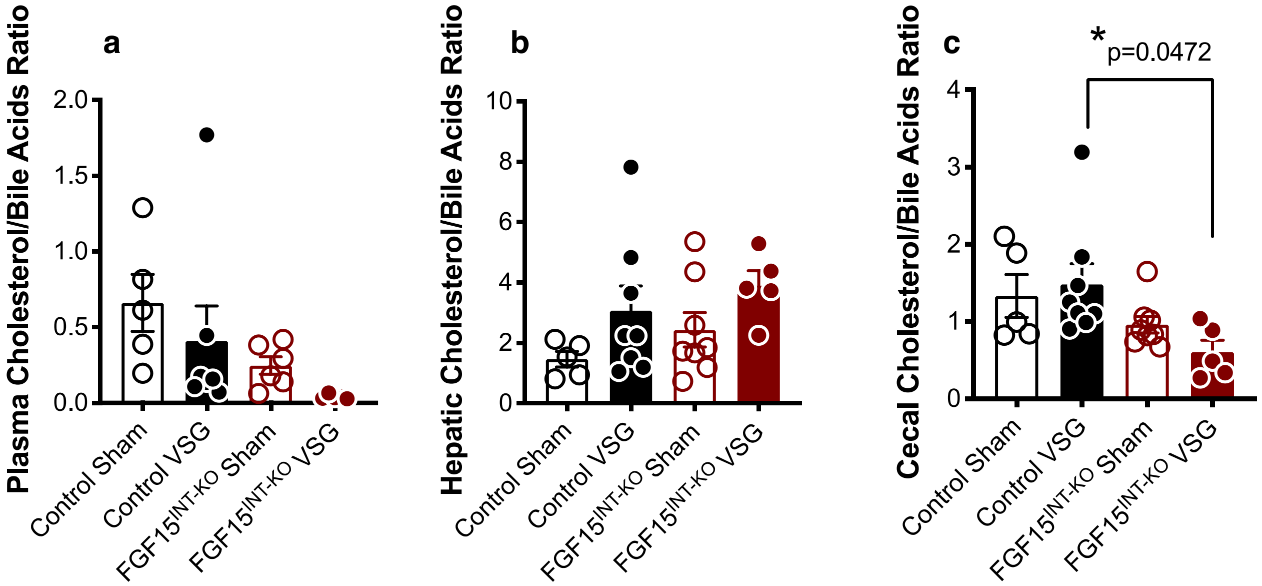

Supplementary Figure 7. Cholesterol to bile acids ratio is altered in FGF15^{INT-KO} mice after VSG. Cholesterol to bile acids ratio in **a.** Plasma, **b.** Liver and **c.** Cecal contents. Animal number for a: Control Sham (n=5), Control VSG (n=7), FGF15^{INT-KO} Sham (n=6), FGF15^{INT-KO} VSG (n=5). Animal number for b and c: Control Sham (n=5), Control VSG (n=8), FGF15^{INT-KO} Sham (n=8), FGF15^{INT-KO} VSG (n=5). Data are shown as means ± S.E.M. *p<0.05 (2-Way ANOVA with Tukey's post-hoc test).

Supplementary Figure 8

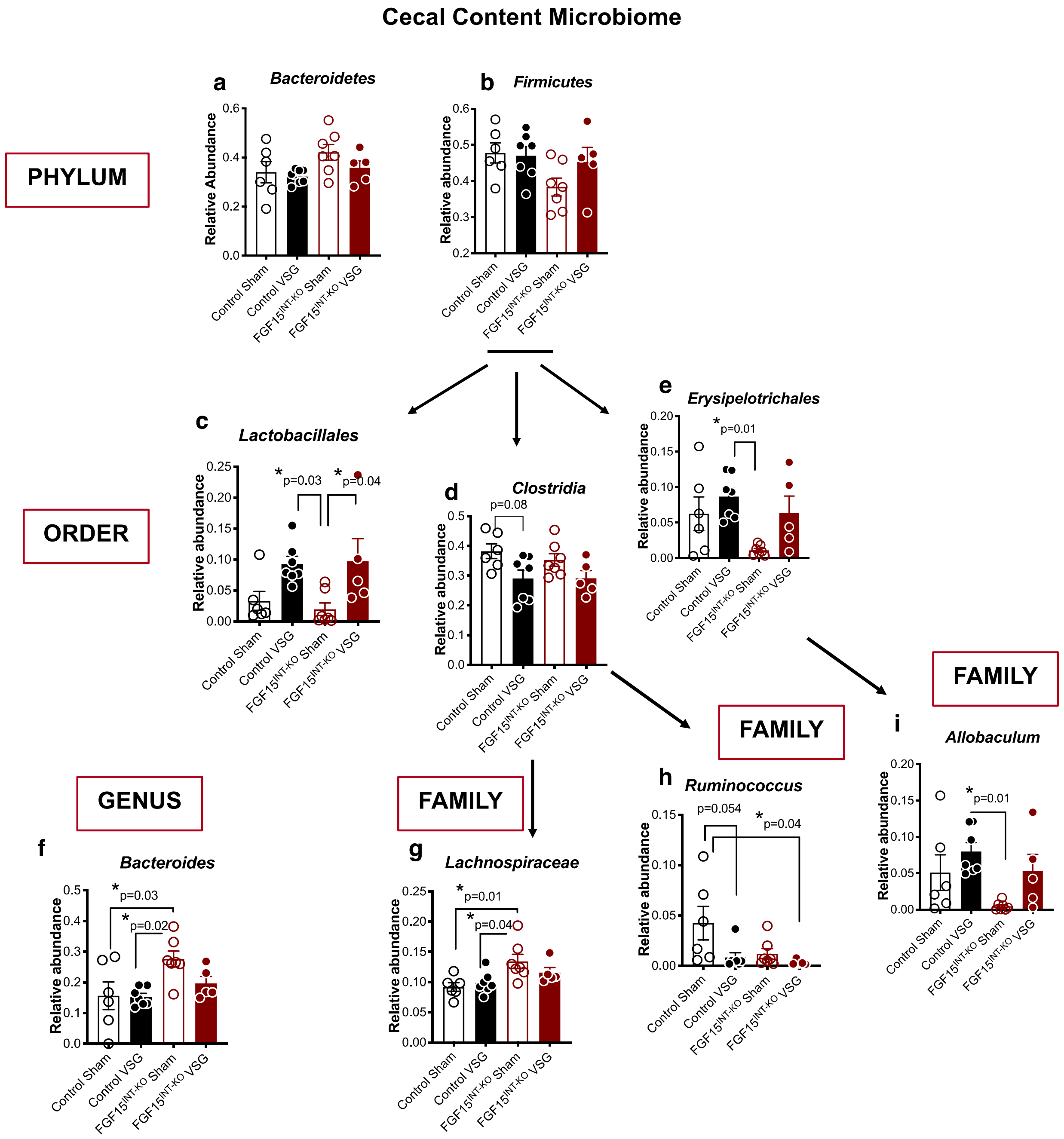

Supplementary Figure 8. Intestinal FGF15 modulates microbiota in cecal content. Relative abundance of **a.** *Bacteroidetes*, **b.** *Firmicutes*, **c.** *Lactobacillales*, **d.** *Clostridales*, **e.** *Erysipelotrichales*, **f.** *Bacteroides*, **g.** *Lachnospiraceae*, **h.** *Ruminococcus*, **i.** *Allobaculum*. Animal number Control Sham (n=6), Control VSG (n=7), FGF15^{INT-KO} Sham (n=7), FGF15^{INT-KO} VSG (n=5). Data are shown as means ± S.E.M. *p<0.05 (2-Way ANOVA with Tukey's post-hoc test).

Supplementary Table 1. Primer source and sequence information

Gene Target	Source	Catalog Number
Primers for mouse *RPL32*	Thermo Fisher Scientific	Cat# Mm02528467_g1
Primers for mouse *Dmt1*	Thermo Fisher Scientific	Cat# Mm00435363_m1
Primers for mouse *Dcytb*	Thermo Fisher Scientific	Cat# Mm01335930_m1
Primers for mouse *Hamp*	Thermo Fisher Scientific	Cat# Mm04231240_s1
Primers for mouse *Slc401a1* (Fpn)	Thermo Fisher Scientific	Cat# Mm01254822_m1
Primers for mouse *HIF2α*	Thermo Fisher Scientific	Cat# Mm01236112_m1
Primers for mouse *Hmcgr*	Thermo Fisher Scientific	Cat# Mm01282499_m1
Primers for mouse *Abcg5*	Thermo Fisher Scientific	Cat# Mm00446241_m1
Primers for mouse *Abcg8*	Thermo Fisher Scientific	Cat# Mm00445980_m1
Primers for mouse *Cyp7a1*	Thermo Fisher Scientific	Cat# Mm00484150_m1
Primers for mouse *Cyp8b1*	Thermo Fisher Scientific	Cat# Mm00501637_s1
Primers for mouse *Cyp27a1*	Thermo Fisher Scientific	Cat# Mm00470430_m1
Primers for mouse *Slc10a1* (Ntcp)	Thermo Fisher Scientific	Cat# Mm00441421_m1
Primers for mouse *Slc1b2* (Oatp4)	Thermo Fisher Scientific	Cat# Mm00451510_m1
Primers for mouse *Slc10a2* (Asbt)	Thermo Fisher Scientific	Cat# Mm00488258_m1
Primers for mouse *Fgf15*	Thermo Fisher Scientific	Cat# Mm00433278_m1
Primers for mouse *Nr1h4* (FXR)	Thermo Fisher Scientific	Cat# Mm00436425_m1
Primers for mouse *Nr0b2* (SHP)	Thermo Fisher Scientific	Cat# Mm00442278_m1
Primers for mouse *FAS*	Thermo Fisher Scientific	Cat# Mm00662319_m1
Primers for mouse *Ppara*	Thermo Fisher Scientific	Cat# Mm00440939_m1
Primers for mouse *Cpt1a*	Thermo Fisher Scientific	Cat# Mm01231183_m1
Primers for mouse *CD36*	Thermo Fisher Scientific	Cat# Mm00432403_m1
Primers for mouse *Fbxo32* (Atrogin 1)	Thermo Fisher Scientific	Cat# Mm00499523_m1
Primers for mouse *Trim63* (MuRF1)	Thermo Fisher Scientific	Cat# Mm01185221_m1
