# Supplementary material for: Intestinal-derived FGF15 protects against deleterious effects of vertical sleeve gastrectomy in mice
Source: Nat Commun. 2021 Aug 6;12:4768. doi: 10.1038/s41467-021-24914-y (PMC8346483; doi:10.1038/s41467-021-24914-y)
Supplement: Supplementary file 2 — Reporting Summary [file 41467_2021_24914_MOESM2_ESM.pdf]

nature research

Corresponding author(s): Randy J. Seeley

Last updated by author(s): 6-2-2021

Reporting Summary

Nature Research wishes to improve the reproducibility of the work that we publish. This form provides structure for consistency and transparency in reporting. For further information on Nature Research policies, see our Editorial Policies and the Editorial Policy Checklist.

Statistics

For all statistical analyses, confirm that the following items are present in the figure legend, table legend, main text, or Methods section.

n/a	Confirmed	
☐	☒	The exact sample size (*n*) for each experimental group/condition, given as a discrete number and unit of measurement
☐	☒	A statement on whether measurements were taken from distinct samples or whether the same sample was measured repeatedly
☐	☒	The statistical test(s) used AND whether they are one- or two-sided<br>*Only common tests should be described solely by name; describe more complex techniques in the Methods section.*
☐	☒	A description of all covariates tested
☐	☒	A description of any assumptions or corrections, such as tests of normality and adjustment for multiple comparisons
☐	☒	A full description of the statistical parameters including central tendency (e.g. means) or other basic estimates (e.g. regression coefficient) AND variation (e.g. standard deviation) or associated estimates of uncertainty (e.g. confidence intervals)
☐	☒	For null hypothesis testing, the test statistic (e.g. *F*, *t*, *r*) with confidence intervals, effect sizes, degrees of freedom and *P* value noted<br>*Give P values as exact values whenever suitable.*
☒	☐	For Bayesian analysis, information on the choice of priors and Markov chain Monte Carlo settings
☒	☐	For hierarchical and complex designs, identification of the appropriate level for tests and full reporting of outcomes
☐	☒	Estimates of effect sizes (e.g. Cohen's *d*, Pearson's *r*), indicating how they were calculated

Our web collection on statistics for biologists contains articles on many of the points above.

Software and code

Policy information about availability of computer code

Data collection	Photos were acquired using Olympus IX73 fluorescence microscopy system (Olympus) and images were analyzed using Olympus cellSens Standard imaging software (Olympus). Grip strength was measured with Columbus Instruments Grip Strength Meter (Colombus Instruments). Fecal energy was assessed by University of Michigan Animal Phenotyping core using Bomb Calorimeter, Parr 6200 and 1108P oxygen bomb. Gene expression was performed by quantitative real time RT-PCR using Taqman gene expression assay (Supplemental Table 1) and was performed using StepOnePlus detection system (Applied Biosystems) with a standard protocol. Pooled amplicon 16S library is then sequenced on the Illumina MiSeq platform using the 500 cycle MiSeq V2 Reagent kit (catalog no. MS-102-2003) according to the manufacturer's instructions with modifications of the primer set with custom read 1/read 2 and index primers added to the reagent cartridge. LC-MS analysis of bile acid composition in plasma was performed on an Agilent system consisting of a 1290 UPLC module coupled with a 6490 Triple Quad (QqQ) mass spectrometer (Agilent Technologies, Santa Clara, CA) operated in MRM mode.
Data analysis	The statistical analysis for comparisons between 2 groups was performed by unpaired (2-tailed) Student's t test. Two-way ANOVA with post hoc Tukey's multiple comparisons post-hoc test was used for comparisons among 4 groups. P values <0.05 were considered significant (GraphPad Prism 8.2.0).<br>Microbiome analysis:Following sequencing, microbiome bioinformatics were run using QIIME 2 2020.2 106. Briefly, non-singleton amplicon sequence variants (ASVs, 100% operational taxonomic units (OTUs)) were generated from raw sequences after trimming with the cutadapt plugin denoising with the dada2 plugin. One Control VSG and one FGF15INT-KO Sham samples were excluded because of low OTUs. Taxonomy was then assigned to ASVs using the classify-sklearn alignment algorithm (Bokulich et al. 2018) against the Greengenes database (Release 13.8) of 99% OTUs reference sequences (McDonald et al. 2012). Alpha diversity metrics including Chao1 and Shannon, which estimate with-in sample richness and diversity respectively, were calculated using the diversity plugin. Chao1 index represents the number of ASVs present in one single sample, while Shannon index accounts for both abundance and evenness of ASVs present. Beta diversity metrics including weighted and unweighted UniFrac distance matrix 107, which estimate between-sample dissimilarity, were scaled and visualized through principle coordinates analysis (PCoA), and further used to determine the significance of the clustering between groups via permutational multivariate analysis of variance (PERMANOVA). Linear discriminant analysis (LDA) effect size (LEfSe) with default parameters 108 and Random Forest

Classifier (QIIME 2 2020.2) with 10-fold cross-validations 109 were computed to identify significantly different microbes in abundance between groups at different taxonomic levels.

For manuscripts utilizing custom algorithms or software that are central to the research but not yet described in published literature, software must be made available to editors and reviewers. We strongly encourage code deposition in a community repository (e.g. GitHub). See the Nature Research guidelines for submitting code & software for further information.

Data

Policy information about availability of data

All manuscripts must include a data availability statement. This statement should provide the following information, where applicable:

- Accession codes, unique identifiers, or web links for publicly available datasets
- A list of figures that have associated raw data
- A description of any restrictions on data availability

Plasma bile acid composition data generated in this study have been deposited in the NIH Common Fund's National Metabolomics Data Repository (NMDR) website, the Metabolomics Workbench, https://www.metabolomicsworkbench.org where it has been assigned Project ID (PR001116). The data can be accessed directly via it's Project DOI: (https://doi.org/10.21228/M8FM51). The 16S rRNA Sequencing composition data generated in this study have been deposited in the Sequence Read Archive (SRA) where it has been assigned BioProject ID PRJNA734599. The data can be accessed directly via it's Project DOI: http://www.ncbi.nlm.nih.gov/bioproject/734599. The authors declare that the data supporting the findings of this study are available within the paper and its supplementary information files. Source data are provided with this paper.

Field-specific reporting

Please select the one below that is the best fit for your research. If you are not sure, read the appropriate sections before making your selection.

☒ Life sciences ☐ Behavioural & social sciences ☐ Ecological, evolutionary & environmental sciences

For a reference copy of the document with all sections, see nature.com/documents/nr-reporting-summary-flat.pdf

Life sciences study design

All studies must disclose on these points even when the disclosure is negative.

Sample size	The sample size was shown to be sufficient to measure the differences between groups for the assay used. The sample size from in vivo studies were determined based on prior experiments and experience, which were used to determine the minimum number of animals needed for evaluating a significance. Animal number Control Sham (n=6), Control VSG (n=8), FGF15INT-KO Sham (n=8), FGF15INT-KO VSG (n=5)
Data exclusions	Animals were euthanized 12 weeks post-surgery. One Control Sham mouse accidentally died during NMR measurements on the day before necropsy. The post-necropsy data on metabolites and tissue gene expression for this Control Sham mouse was excluded, but the data not sensitive to nutritional state was included (body composition, etc). One Control VSG and one FGF15INT-KO Sham samples were excluded from microbiome analysis because of low OTUs generated for these samples. Plasma bile acid composition analysis identified 3 samples that were abnormally elevated. Using ROUT method and treating all the values in all subgroups as one set of data (Q=1), we identified the 3 samples (one Control VSG and two FGF15INT-KO Sham mice) as significant outliers. QQ plot and Homoscedasticity plot showed that these samples were out of the normal distribution. Therefore, these samples were excluded from all plasma bile acid data analysis. One Control VSG ileum sample was excluded due to high cycle threshold of RL32 (Ct over 30 after two independent measurements).
Replication	All metabolic studies were performed at least twice with same results and data was shown over multiple time points (body weight, body composition, food intake, glucose tolerance). All replication attempts were successful and observed metabolic effects of VSG in control/WT mice were consistent with previous observations and published results.
Randomization	Samples and animal order were randomized
Blinding	Investigators were blinded during data collection

Reporting for specific materials, systems and methods

We require information from authors about some types of materials, experimental systems and methods used in many studies. Here, indicate whether each material, system or method listed is relevant to your study. If you are not sure if a list item applies to your research, read the appropriate section before selecting a response.

Materials & experimental systems

n/a	Involved in the study
x	Antibodies
x	Eukaryotic cell lines
x	Palaeontology and archaeology
	x Animals and other organisms
x	Human research participants
x	Clinical data
x	Dual use research of concern

Methods

n/a	Involved in the study
x	ChIP-seq
x	Flow cytometry
x	MRI-based neuroimaging

Animals and other organisms

Policy information about studies involving animals; ARRIVE guidelines recommended for reporting animal research

Laboratory animals	FGF15 flox/flox mice, Villin Cre-ERT2 mice, C57BL background, male mice, 6-8 weeks old at initiation of studies, 26-28 weeks old at end of studies. Mice were singly-housed under a 12-hour light/dark cycle in a facility maintained at 25°C with 50-60% humidity.
Wild animals	No wild animals were used in this study
Field-collected samples	No field-collected samples were used in this study
Ethics oversight	All protocols complied with all relevant ethical regulations for animal testing and research. All protocols were approved by the University of Michigan (Ann Arbor, MI) Animal Care and Use Committees and were in accordance to NIH guidelines.

Note that full information on the approval of the study protocol must also be provided in the manuscript.
